# The economic impact of COVID-19 interventions: A mathematical modeling approach

**DOI:** 10.3389/fpubh.2022.993745

**Published:** 2022-09-12

**Authors:** Jung Eun Kim, Heejin Choi, Yongin Choi, Chang Hyeong Lee

**Affiliations:** ^1^Department of Mathematical Sciences, Ulsan National Institute of Science and Technology, Ulsan, South Korea; ^2^Busan Center for Medical Mathematics, National Institute of Mathematical Sciences, Daejeon, South Korea

**Keywords:** COVID-19, mathematical model, cost estimation, vaccination, social distancing

## Abstract

Prior to vaccination or drug treatment, non-pharmaceutical interventions were almost the only way to control the coronavirus disease 2019 (COVID-19) epidemic. After vaccines were developed, effective vaccination strategies became important. The prolonged COVID-19 pandemic has caused enormous economic losses worldwide. As such, it is necessary to estimate the economic effects of control policies, including non-pharmaceutical interventions and vaccination strategies. We estimated the costs associated with COVID-19 according to different vaccination rollout speeds and social distancing levels and investigated effective control strategies for cost minimization. Age-structured mathematical models were developed and used to study disease transmission epidemiology. Using these models, we estimated the actual costs due to COVID-19, considering costs associated with medical care, lost wages, death, vaccination, and gross domestic product (GDP) losses due to social distancing. The lower the social distancing (SD) level, the more important the vaccination rollout speed. SD level 1 was cost-effective under fast rollout speeds, but SD level 2 was more effective for slow rollout speeds. If the vaccine rollout rate is fast enough, even implementing SD level 1 will be cost effective and can control the number of critically ill patients and deaths. If social distancing is maintained at level 2 at the beginning and then relaxed when sufficient vaccinations have been administered, economic costs can be reduced while maintaining the number of patients with severe symptoms below the intensive care unit (ICU) capacity. Korea has wellequipped medical facilities and infrastructure for rapid vaccination, and the public's desire for vaccination is high. In this case, the speed of vaccine supply is an important factor in controlling the COVID-19 epidemic. If the speed of vaccination is fast, it is possible to maintain a low level of social distancing without a significant increase in the number of deaths and hospitalized patients with severe symptoms, and the corresponding costs can be reduced.

## Introduction

The outbreak of coronavirus disease 2019 (COVID-19) occurred in 2019, and it is still affecting the world in 2022. When there was no effective vaccine or treatment in the early stage of COVID-19, the only control strategy against the disease involved non-pharmaceutical interventions, such as social distancing and lockdowns.

The method of implementing these non-pharmaceutical interventions varied from country to country, but most such strategies have had a significant effect in reducing the number of confirmed cases, severe cases, and deaths. However, as the spread of COVID-19 became prolonged, the economic damage caused by the non-pharmaceutical interventions increased significantly. Thus, when implementing an effective control policy, it is very important to estimate not only the effect on the reduction of confirmed cases, severe cases, and deaths, but also the economic damage.

In Korea, the vaccination rate has steadily increased since vaccination began on 26 February 2021 and, as of June 2022, 86% of the total population had completed their second vaccination, while the number with a third vaccination has reached 65% of the total population (1). However, when the vaccines were being introduced, vaccination was not implemented quickly due to supply restrictions, and the number of vaccinations only increased rapidly after June 2021. Koreans have a favorable attitude toward vaccination and possess the infrastructure for large-scale vaccination. This study investigates the effect of the vaccination rollout speed at the beginning of vaccination, based on various scenarios.

Estimation of the time-dependent dynamics of the number of confirmed cases, severe cases, and deaths due to COVID-19, as well as economic damage, can be conducted using mathematical modeling, which has been previously used to describe the transmission of COVID-19. Based on an age-structured compartmental model, the effects of non-pharmaceutical interventions (NPIs) and vaccination policies have been studied (2–4). Estimation of the number of confirmed cases has been investigated through simulation of various scenarios based on the vaccination plan and social distancing (5–7). In some studies (8, 9), the cost-effectiveness of control strategies against COVID-19 has been studied through the use of mathematical models. As the vaccines against COVID-19 were developed at the end of 2020, cost-effective control including vaccination has become important and cost-effective control strategies based on different vaccine allocation policies have been studied (10, 11). There have been studies (12–14) using mathematical models that considered mild, hospitalized, and critical symptom cases without an age structure. However, since the important parameters of COVID-19 such as transmission rate, severity and death rate are closely related to age (1, 7, 15), it is necessary to apply an age-structured model for analyzing the effect of the transmission and control of the disease. In order to study the economic effect of COVID-19, we constructed a mathematical model considering both the age-specific structure and the subdivided severity.

In this paper, we use age-specific mathematical models to estimate the cost of COVID-19 in terms of medical expenses, wage loss, cost due to deaths, vaccination cost, and gross domestic product (GDP) loss. Efficient policies, according to vaccination rollout speed and social distancing levels, are investigated by considering both cost reduction and the control of the number of patients with severe symptoms. We also investigate the mitigation effect of social distancing policies on the total cost due to COVID-19 and the number of critically ill patients.

## Methods

### Epidemiological data

**Figure 1:**
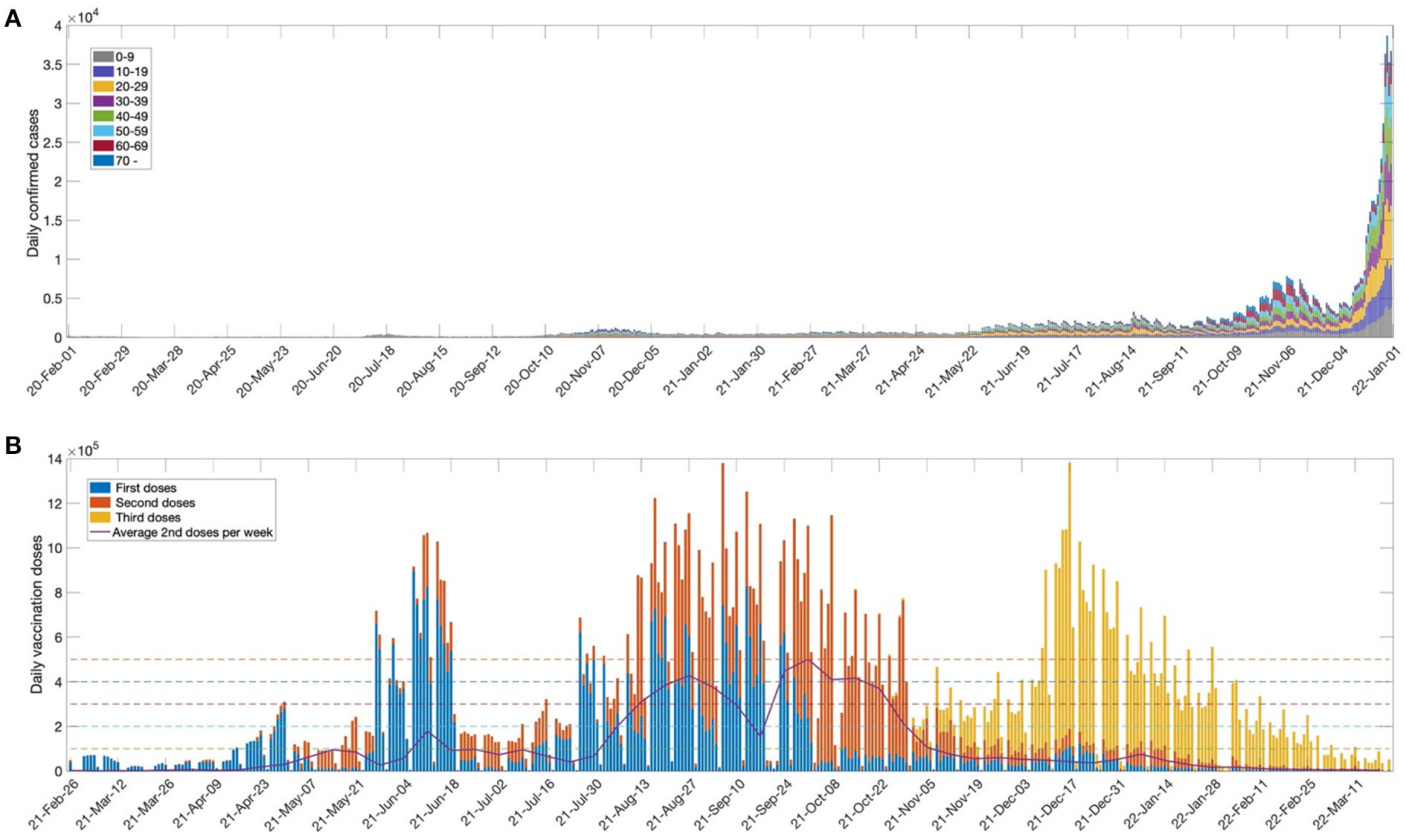
**(A)** Number of weekly confirmed cases for age groups (0–9,10–19,20–29,30–39,40–49,50–59,60–69, 70+) and **(B)** Daily first, second and third vaccination doses. The solid curve indicates average second doses per week. The horizontal dashed lines represent daily vaccination doses (ν=100000, 200000, . . , 500000) used in the model simulation.

Since the first confirmed case was reported on 19 January 2020, Korea has steadily reported cases of COVID-19. Figure 1A shows the daily number of confirmed cases, by age group, from 1 February 2020 to 31 December 2021. In November 2021, the vaccination coverage rate approached 80% of the total population, and the social distancing phase-easing policy was implemented on 1 November 2021. However, this resulted in a significant increase in the daily number of confirmed cases. In particular, the number of confirmed cases in patients under the age of 18 who were not vaccinated increased significantly. In addition, the number of confirmed cases has increased rapidly due to the prevalence of the Omicron variant and waning vaccine-based immunity. As of 17 March 2022, the number of daily confirmed cases exceeded 600,000 (1). Figure 1B shows the first, second, and third vaccination doses per day from 26 February 2021, when the vaccination program was started in Korea. It shows that, until May 2021, the daily dose was relatively small, due to the limited vaccine supply. A significant increase in the number of vaccinations was observed after July 2021, which can be seen as indicating the favorable reception of the Korean public for vaccination and the sufficient vaccination infrastructure. The third vaccination was also actively promoted and, as of May 2022, more than 65% of the population received booster shots (1).

In this work, we investigated the importance of the vaccination rollout speed for disease outbreak control. The horizontal dashed lines in the figure indicate the daily dose (ν = 100000, 200000, …, 500000) used for model simulation. The maximum daily dose (ν = 500,000) was set based on the average weekly second vaccination doses in Korea. We also considered the social distancing levels implemented in Korea in order to determine the effect of non-pharmaceutical intervention policies. Details of the social distancing polices used in Korea are given in Supplementary material section 1.

### Mathematical model

**Figure 2:**
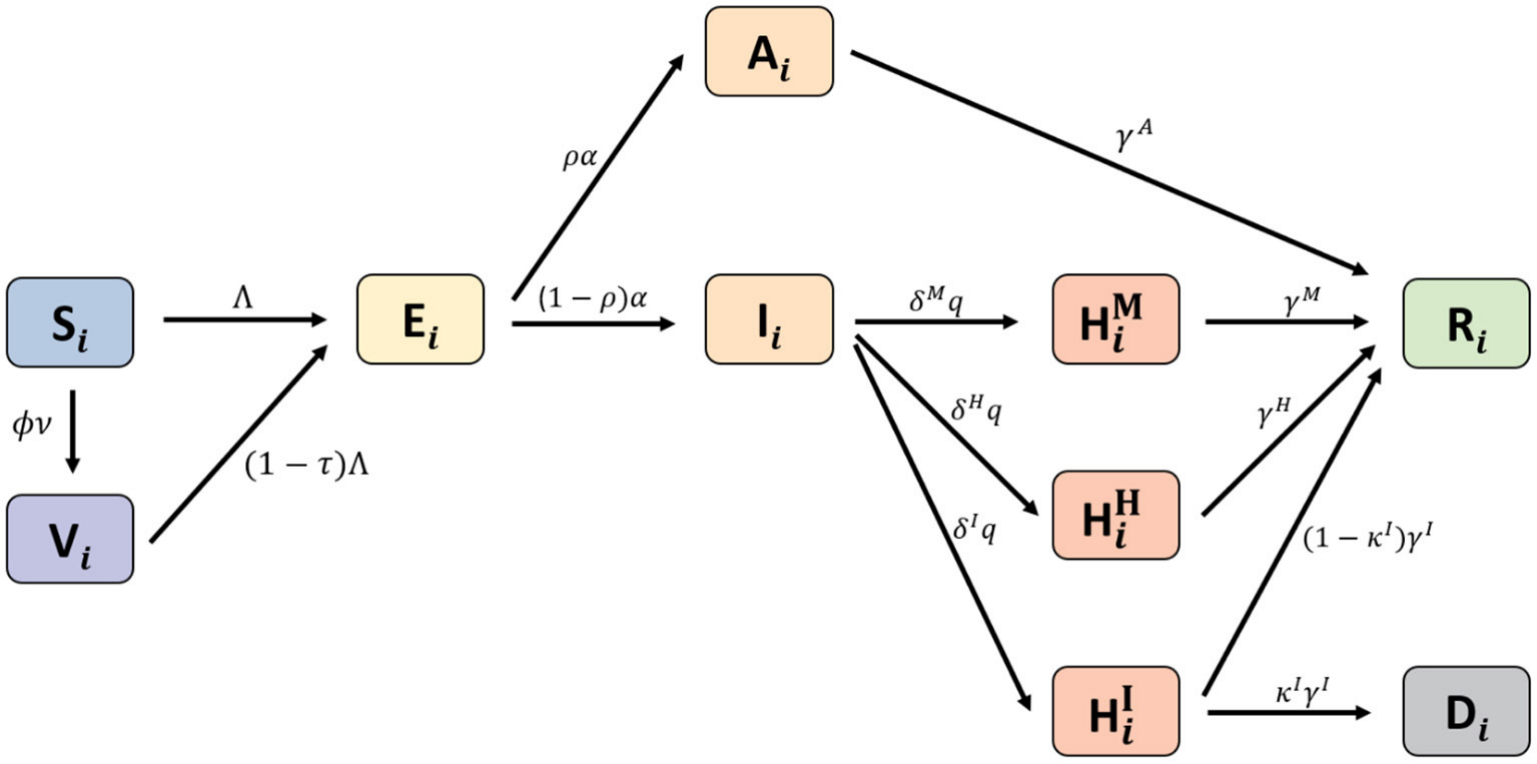
Schematic diagram for the proposed model.

We developed an age-structured mathematical model to describe the transmission dynamics of COVID-19 with vaccination. In the model, the population was separated into compartments, based on their characteristics, for each age group *i*: *S*_*i*_(*t*) denotes susceptible, *V*_*i*_(*t*) denotes completely vaccinated, *E*_*i*_(*t*) denotes exposed, *A*_*i*_(*t*) denotes asymptomatic, *I*_*i*_(*t*) denotes infectious, $H_{i}^{M}\left(t\right)$ denotes having mild symptoms, $H_{i}^{H}\left(t\right)$ denotes hospitalized without intensive care, $H_{i}^{I}\left(t\right)$ denotes hospitalized with intensive care, *R*_*i*_(*t*) denotes recovered, and *D*_*i*_(*t*) denotes deceased. The *H*^*M*^, *H*^*H*^, and *H*^*I*^ groups correspond to the highest severity level of an individual during the quarantine period. The age classes *i* = 1, 2, ⋯ , 8 represent individuals aged 0−9, 10−19, 20−29, 30−39, 40−49, 50−59, 60−69, and older than 70 years, respectively. A schematic diagram of the model is shown in Figure 2. The system of differential equations that describes the model dynamics is as follows:


$$\begin{array}{l} \dot{S_{i}}\;=-{\text{Λ}}_{i}S_{i}-\phi_{i}\nu \;\\ \dot{V_{i}}\;=\phi_{i}\nu -\left(1-\tau \right){\text{Λ}}_{i}V_{i}\\ \dot{E_{i}}\;={\text{Λ}}_{i}S_{i}+\left(1-\tau \right){\text{Λ}}_{i}V_{i}-\alpha E_{i}\;\\ \dot{A_{i}}\;=\text{ρα}E_{i}-\gamma^{A}A_{i}\;\\ \dot{I_{i}}\;=\left(1-\rho \right)\alpha E_{i}-qI_{i}\;\\ \dot{H_{i}^{M}}=q\delta_{i}^{M}I_{i}-\gamma_{i}^{M}H_{i}^{M}\;\\ \dot{H_{i}^{H}}=q\delta_{i}^{H}I_{i}-\gamma_{i}^{H}H_{i}^{H}\;\\ \dot{H_{i}^{I}}=q\delta_{i}^{I}I_{i}-\left(1-\kappa_{i}^{I}\right)\gamma_{i}^{I}H_{i}^{I}-\kappa_{i}^{I}\gamma_{i}^{I}H_{i}^{I}\;\\ \dot{R_{i}}\;=\gamma^{A}A_{i}+\gamma_{i}^{M}H_{i}^{M}+\gamma_{i}^{H}H_{i}^{H}+\left(1-\kappa_{i}^{I}\right)\gamma_{i}^{I}H_{i}^{I}\;\\ \dot{D_{i}}\;=\kappa_{i}^{I}\gamma_{i}^{I}H_{i}^{I} \end{array}$$

Where the force of infection for age group *i* is obtained by $\Lambda_{i}=\underset{j}{\sum }\left[\beta_{ij}\left(I_{j}+\theta_{A}A_{j}\right)/N_{j}\right]\;$ with the disease transmission rates β_*ij*_, for *N*_*j*_ = *S*_*j*_+*V*_*j*_+*E*_*j*_+*A*_*j*_+*I*_*j*_+*R*_*j*_.

The disease transmission rates (β_*ij*_) of a person in age group *i* per contact for each age group *j* are estimated as 8 × 8 matrices, determined by minimizing the difference between confirmed case data and the simulated results from the model (in the least-squares sense) for social distancing levels (LV) 0, 1, 2, and 3 in Korea. The transmission rate matrices and the data-fitting results for all ages are provided in the Supplementary material section 2. Other parameters used in the model and the baseline values are given in Table 1.

**Table 1:** Parameter definitions and baseline values used in numerical simulations.

**Parameter**	**Description**	**Value**	**Reference**
Λ_*i*_	The force of infection for age group *i*		Estimated
β_*ik*_	Transmission rate from age group *k* to *i*	Given in Supplementary material section 2	Estimated
ϕ_*i*_	Vaccination allocation vector	Vary	
ν	Daily vaccination doses	Vary	
τ	Vaccine efficacy	0.79, vary	(7)
ρ	Probability of unconfirmed asymptomatic cases	0.16	(7)
1/α	Latent period	5.2	(16)
${\delta_{i}}^{M}$	Probability of cases having mild symptoms	1, 0.996, 0.991, 0.984, 0.971, 0.924, 0.854, 0.694	(15)
${\delta_{i}}^{H}$	Probability of hospitalization without intensive care	0, 0.002, 0.007, 0.012, 0.027, 0.068, 0.123, 0.275	(15)
${\delta_{i}}^{I}$	Probability of hospitalization with intensive care	$1-{\delta_{i}}^{M}{{-\;\delta }_{i}}^{H}$	
1/*q*	Mean duration of the case confirmation	3	(6)
1/γ^*A*^	Recovery period of asymptomatic cases	3.5	(7)
1/γ^*M*^	Recovery (or quarantine) period of mild symptom cases	14 (treatment center) 7 (home treatment)	(1)
$1/\gamma_{i}^{H}$	Recovery period of hospitalized without intensive care cases	15.32, 15.99, 18.66, 17.70, 17.84, 18.44, 19.77, 23.79	(1)
$1/{\gamma_{i}}^{I}$	Recovery period of hospitalized with intensive care cases	15.32, 15.99, 18.66, 17.70, 17.84, 18.44, 19.77, 23.79	(1)
μ_*i*_	Death rate of groups in confirmed cases	0, 0, 0.0001, 0.0004,0.0007, 0.0027, 0.0108, 0.0513	(17)
*r* ^*H*^	Probability of death from hospitalization without intensive care	0.4216	Estimated
*r* ^*I*^	Probability of death from hospitalization with intensive care	0.5786	Estimated
κ^*I*^	Mortality rate of hospitalization with intensive care cases	μ/δ^*I*^	
θ_*A*_	Relative infectiousness of asymptomatic infections	0.51	(18)

The effective reproduction number (*R*_*t*_) measures the average number of secondary cases per infectious individual at time t, which is obtained by calculating the spectral radius of the next-generation matrix. The details of the derivation of *R*_*t*_ of the model are given in Supplementary material section 3.

### Cost estimation

The total cost due to COVID-19 was estimated in terms of medical expenses, wage loss, cost due to deaths, vaccination cost, and the GDP loss due to implementing the social distancing polices. The cost of medical expenses was computed considering the average daily cost of treatment and the recovery period with respect to the status of patients (i.e., mild symptom cases, hospitalized cases without intensive care, or hospitalized cases with intensive care). In Korea, before 10 February 2022, even patients with mild symptoms were admitted to community treatment centers and quarantined; however, after that, the policy was changed to give priority to home treatment. As a result, the quarantine period and cost decreased. The costs according to these two policies were calculated and compared. The cost of wage loss for patients older than 20 years was computed considering the average daily income, the employment rate, and the recovery period for each age group. The cost of wage loss for patients younger than 19 years was computed as the income decrease for females with infected children younger than 19 years. The cost due to death was calculated by summing the average funeral cost per capita and the present value of the predicted future income for the potential economic production loss using the forgone labor output equation (19). The vaccination cost included the average vaccine price, the procedure cost, and the logistics cost. The GDP loss was estimated by multiplying the GDP reduction rate according to the SD level with the 2019 GDP. The formulae for the estimation of each factor of the total cost are given in Table 2, and the parameters used for cost estimation are given in Table 3.

**Table 2:** Formulae for the cost estimation.

**Cost factor**	**Formula**
**Medical expenses**	
Mild symptom case (*H*^*M*^)	$\sum_{i=1}^{8}[$ Average daily cost of treatment for mild patients (*C*_*M*_) × Recovery period of mild symptom cases $\left(1/\gamma_{i}^{M}\right)\times$ Number of mild patients $\left(H_{i}^{M}\right)$]
Hospitalized case without intensive care (*H*^*H*^)	$\sum_{i=1}^{8}[$ Average daily cost of treatment for hospitalized patients without intensive care (*C*_*H*_) × Recovery period of hospitalized patients without intensive care $\left(1/\gamma_{i}^{H}\right)\times$ Number of hospitalized patients without intensive care $\left(H_{i}^{H}\right)$]
Hospitalized case with intensive care (*H*^*I*^)	$\sum_{i=1}^{8}[$ Average daily cost of treatment for hospitalized patients with intensive care (*C*_*I*_) × Recovery period of hospitalized patients with intensive care $\left(1/\gamma_{i}^{I}\right)\times$ Number of hospitalized patients with intensive care $\left(H_{i}^{I}\right)$]
**Wage loss**	
Older than 20 years	$\sum_{x=M,H,I}[\sum_{i=1}^{8}[$ Average daily income in age group *i* (*W*_*i*_) × Employment rate in age group *i* (*E*_*i*_) × Recovery period of cases $\left(1/\gamma_{i}^{x}\right)\;\times$ Number of patients $\left(H_{i}^{x}\right)$]]
Younger than 19 years	Average daily income of women in their 30s and 40s (*W*_*f*_) × Female employment rate of children younger than 19 years (*E*_*f*_) × Average recovery period × Number of patients younger than 19 years
**Death**	Average funeral cost (FC)+Present value of the predicted future income (PV)$\left(PV=\overset{N_{i}}{\underset{j=1}{\sum }}\frac{W_{j}{\left(1+g\right)}^{j}}{{\left(1+r\right)}^{j}}\right)$
**Vaccination cost**	[Vaccination cost per person (VC) + Vaccination procedure cost (PC) + Logistics cost (LC)] × Population × Vaccination rate (VR)
**GDP loss**	GDP loss rate for social distancing level *j* (*GDP*_*los*_*s*__*j*__) × GDP in 2019 × Simulation period (year)

**Table 3:** Descriptions and values of parameters for cost estimation.

	**Description**	**Value**	**Reference**
*C*_*M*_	Medical cost for mild patients per day	$160.8 (treatment center) $5 (home treatment)	(20, 21)
*C*_*H*_	Medical cost for number of hospitalized patients without intensive per day	$432.5	(20, 21)
*C*_*I*_	Medical cost for hospitalized patients with intensive per day	$1,129.5	(20, 21)
*E*_*i*_	Employment rate in age group *i*	0, 04, 0.557, 0.753, 0.771, 0.743, 0.566, 0.23	(22)
*E*_*f*_	Female employment rate with children younger than 19 years	0.555	(22)
*W*_*i*_	Average daily income in age group *i*	0, 55.47, 76.63, 109.40, 129.33, 127.14, 82.14, 68.08	(23)
*W*_*f*_	Average daily income of women in 30s and 40s	99.3500	(23)
*g*	Average annual salary increase rate	0.02	(24)
*r*	Social discount rate	0.04	(24)
*N*_*i*_	Average working period in age group *i*	50, 50, 45, 35, 25, 15, 5, 0	Assumed
FC	Average funeral cost	$9,405.38	(25)
VC	Vaccination cost per person	$17.89	(26)
PC	Vaccination procedure cost	$16.87	(27)
LC	Logistics cost	$1	Assumed
VR	Vaccination rate	0.8	Assumed
GDP	GDP per capita in 2019	$31,929	(28)
*GDP*_*loss*_*j*__	GDP loss rate for social distancing level *j* = 1, 2, 3	0.002, 0.018, 0.064	(29)

## Results

### The effect of rollout speed of vaccination on disease transmission

The effects of the daily vaccination doses on the number of confirmed cases, cumulative deaths, and hospitalized population with intensive care unit (ICU) care were investigated through numerical simulation using the mathematical model. As the COVID-19 vaccination program began on 26 February 2021, we set the initial date of the simulation as 1 April 2021, and the duration of the simulation was set as 365 days. The initial conditions were determined with respect to the confirmed case data in Korea at the start date of the simulation (1). At the beginning of vaccination in Korea, vaccination was implemented for those aged 19 years and older; however, in October 2021, the vaccine target was expanded to those aged 12 years and older.

**Figure 3:**
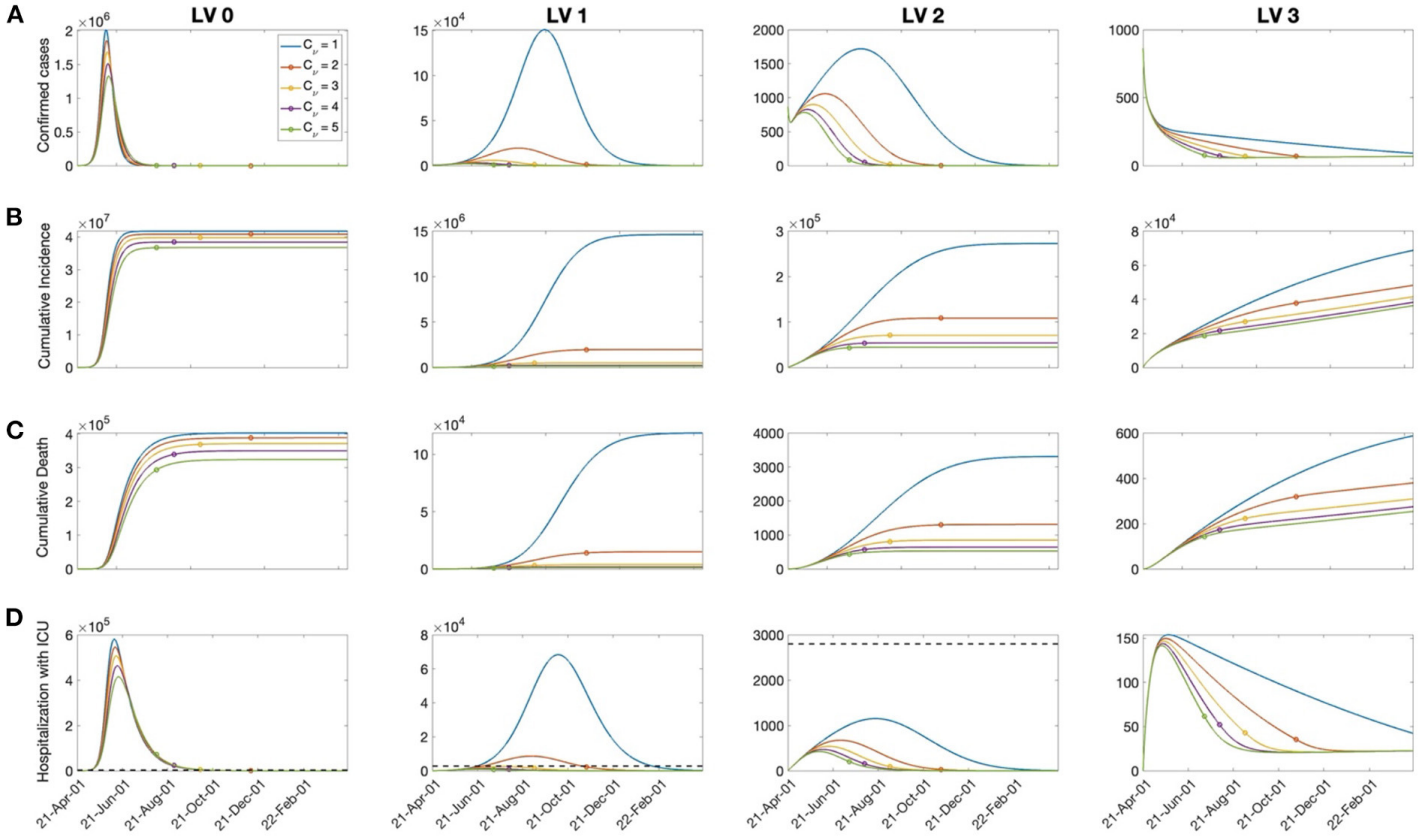
The effects of vaccination on: **(A)** Confirmed cases, **(B)** cumulative confirmed cases, **(C)** death, and **(D)** hospitalized population in the ICU for C_ν_=1,2,⋯, 5 and the SD level 0, 1, 2, 3. The dashed line in the bottom panels represents the capacity of ICU bed for COVID-19 patients in Korea. The circle on each curve represents the time at which the vaccination coverage rate reaches 80%.

In this work, it was assumed that vaccination was administered to those aged 10 years or older, and vaccination was terminated when 80% of the total population of Korea was vaccinated. It was assumed that inoculation was evenly distributed according to the number of people in each age group. We used ν_0_ = 100, 000 as the baseline value for the daily vaccination dose, based on the average daily completed vaccinations in Korea (see Figure 1B). The daily vaccination dose varied as ν = *C*_ν_ν_*o*_ for *C*_ν_ = 1, 2, ⋯ , 5. Figure 3 shows the time-series of daily new confirmed cases, cumulative confirmed cases, cumulative deaths, and the hospitalized population in intensive care, with respect to the rollout speed (*C*_ν_ = 1, 2, ⋯ , 5) under SD levels 0, 1, 2, and 3. It can be seen that, under SD levels 1 and 2, the rollout speed significantly affected the reduction in the number of confirmed cases. Therefore, when a moderate level of social distancing is implemented, the speed of vaccine release becomes even more important. It has been reported that the number of available ICU beds for COVID-19 patients in Korea is about 2800, as indicated by the dashed lines in Figure 3D (17). Figure 3D shows that, under SD LV0 and LV1 with slow vaccination speed (*C*_ν_ = 1, 2), the maximum number of ICU patients exceeds 2800, thus posing a burden on the Korean medical system. The numbers of cumulative confirmed cases, cumulative death, and maximum hospitalized population in ICU for various social distancing level, rollout speed (*C*_ν_ = 1, 2, ⋯ , 5), and vaccine efficacy (τ = 0.79, 0.6) are given in Supplementary material section 4.

### Cost estimation due to COVID-19

**Figure 4:**
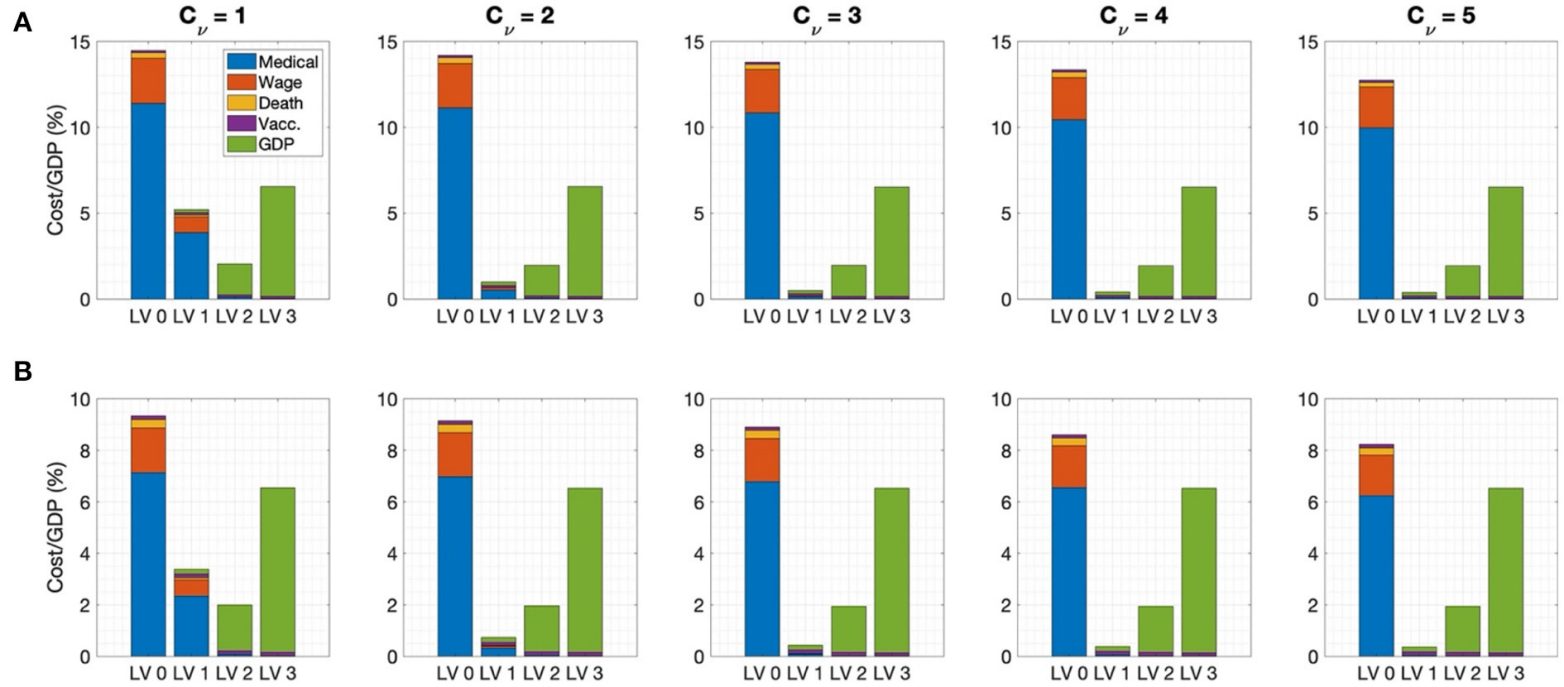
Ratio of cost to GDP for C_ν_=1, 2, ⋯, 5 for **(A)** admission to the treatment center for mild patients **(B)** home treatment for mild patients.

The cost due to COVID-19 was estimated based on medical costs, wage losses, deaths costs, immunization costs, and GDP losses under various social distancing policies considering different rollout speeds and SD levels. Figure 4 shows the cost of each factor calculated over a one-year period with rollout speed *C*_ν_ = 1, 2, ⋯ , 5 and SD level 0, 1, 2, 3 under (A) treatment center admission and (B) home treatment for patients with mild symptoms. When the rollout rate was low (*C*_ν_ = 1), implementing SD LV 2 was the least costly; otherwise, SD LV 1 was more cost effective. The medical expenses accounted for the largest proportion of the total cost at SD level 0 or 1, while the GDP loss accounted for the largest proportion at SD level 2 or 3. Home treatment, instead of being admitted to a treatment center, for the patients having mild symptoms reduces medical costs by about 40%. The cost values for each factor are given in the Supplementary material section 5. If the rollout speed is fast, the number of fatalities and hospitalizations with severe symptoms is not significantly different under SD levels 1 and 2. Therefore, increasing the vaccine supply rate can bring economic cost savings while maintaining a low SD level, without a significant increase in the number of fatalities and patients with severe symptoms.

### The effects of social distancing level mitigation

As the vaccination rate in Korea increased and the economic and psychological damage to people caused by the long-term implementation of social distancing strengthened, the necessity for mitigating the social distancing level emerged. However, the number of confirmed cases surged as social distancing was eased in accordance with the “Reorganization for the Step-by-step Daily Recovery” implemented in November 2021 in Korea.

**Figure 5:**
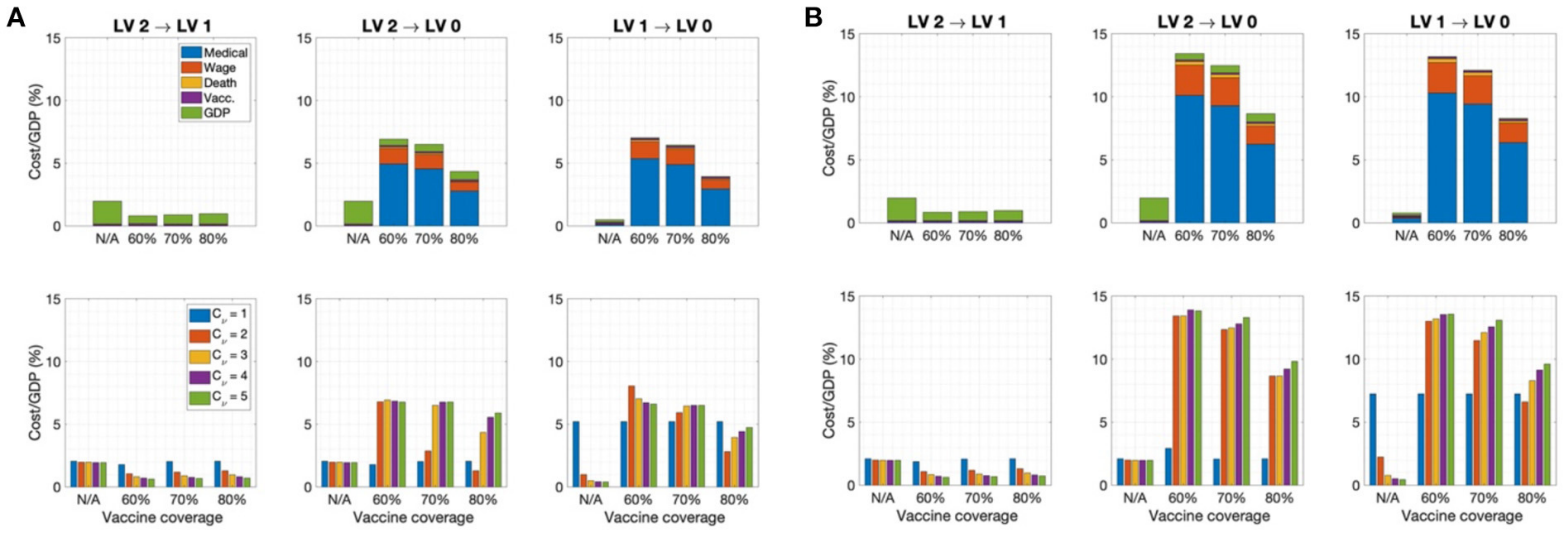
Ratio of cost to GDP for each SD level mitigation scenario when the vaccination coverage rate reaches 60, 70, or 80% in case of admission to the treatment center for mild patients for **(A)** τ = 0.79 and **(B)** τ = 0.6 for (Top) *C*_ν_ = 3. (Bottom) *C*_ν_ = 1, 2, ..., 5. (N/A indicates that social distancing easing was not implemented).

In this section, the economic effects of social distancing mitigation policies are investigated. Social distancing mitigation scenarios were assumed as a relaxation from SD LV 2 to LV 1, SD LV 2 to LV0, or SD LV 1 to LV 0 when the vaccination rate of the total population reached 60, 70, or 80%. Figure 5 shows the cost of each factor for each social distancing mitigation scenario in the case of admission to a treatment center for patients with mild symptoms in the top panel for *C*_ν_ = 3 , and the total cost for *C*_ν_ = 1, 2, ..., 5 *in the bottom panel*. In addition, cases with (A) high vaccine efficiency (τ = 0.79) and (B) low vaccine efficiency (τ = 0.6) were compared.

It can be seen that the most cost-effective scenario was to implement SD mitigation from LV2 to LV1 when the vaccination rate reached 60%. At this time, there was no significant increase in the number of deaths and the number of patients with severe symptoms. However, with SD LV2 to LV0 mitigation in the early stage, the cost increased significantly. In particular, the lower the vaccine efficiency, the greater the cost due to early mitigation. Figure 5 shows that, in the SD LV2 to LV0 and LV2 to LV1 cases, the faster the rollout speed, the higher the total cost under the same vaccine coverage. Therefore, it can be seen that a certain level of social distancing is necessary even if vaccination has sufficiently progressed. A time-series of the number of confirmed cases under each SD level mitigation scenario is provided in the Supplementary material section 6.

### The vaccination effects on SARS-CoV-2 variants

It has been shown that vaccine efficacy may decrease due to COVID-19 variants, such as Omicron, and waning vaccine-based immunity (30–32). In addition, the disease trans-mission rate can change depending on the characteristics of the variant and non-pharmaceutical intervention policies. In this section, we investigate the effects on the total cost due to COVID-19 and number of hospitalized patients in the ICU due to changes in vaccine efficacy and the disease transmission rate.

**Figure 6:**
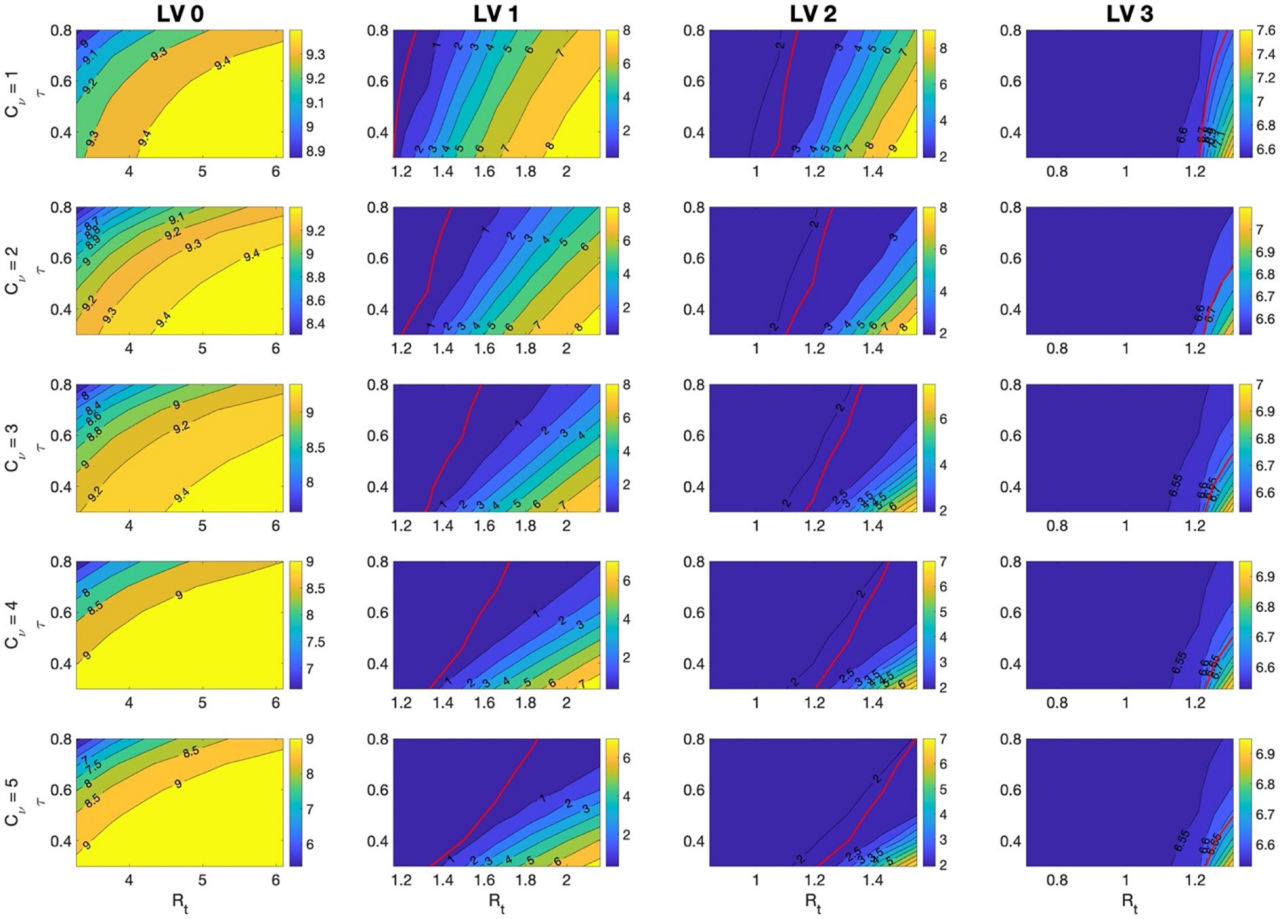
The ratio of total costs to GDP for τ=0.3 − 0.8 and for R_t_ corresponding to β that is varied as β × C_β_ for C_β_=0.7 − 1.3 under SD LV 0, 1, 2, and 3, and the rollout speed C_ν_=1, 2, 3, 4, 5. Red lines indicate the case when the maximum number of hospitalized patients with severe symptoms is the capacity of the intensive care unit, 2,800, for COVID-19 patients in Korea. It means that the maximum number hospitalized patients with severe symptoms is lower than capacity on the left side of the red curves.

In Figure 6, the total costs are computed for the vaccine efficacy varying from τ = 0.3−0.8 and for the *R*_*t*_ corresponding to the transmission rate matrix β, which changes as β × *C*_β_for *C*_β_ = 0.7−1.3 under SD LV 0, 1, 2, and 3, and the rollout speed *C*_ν_ = 1, 2, …, 5. The figure also represents the cases where the maximum number of hospitalized patients with severe symptoms is greater than the capacity of the intensive care unit for COVID-19 patients in Korea, shown as red curves.

It was found that, under SD LV 1 and 2, the total cost changed more sensitively with changes in τ and *R*_*t*_. In these cases, the total cost and the number of hospitalized patients with severe symptoms were also greatly affected by changes in the rollout speed. The number of hospitalized patients with severe symptoms depended more on the change in *R*_*t*_ than on the change in τ. If the vaccine efficiency is high and the rollout speed is sufficiently fast, the number of hospitalized patients with severe symptoms can be kept below the ICU bed capacity, even under SD LV 1. When the vaccine efficacy τ is low, the effect of a change in β on the total cost is greater. Therefore, the less effective the vaccine, the more effort is needed to reduce the transmission rate of the disease. The cumulative number of confirmed cases and the maximum number of hospitalized patients with severe symptoms in each case are included in the Supplementary material section 7.

### Sensitivity analysis

**Figure 7:**
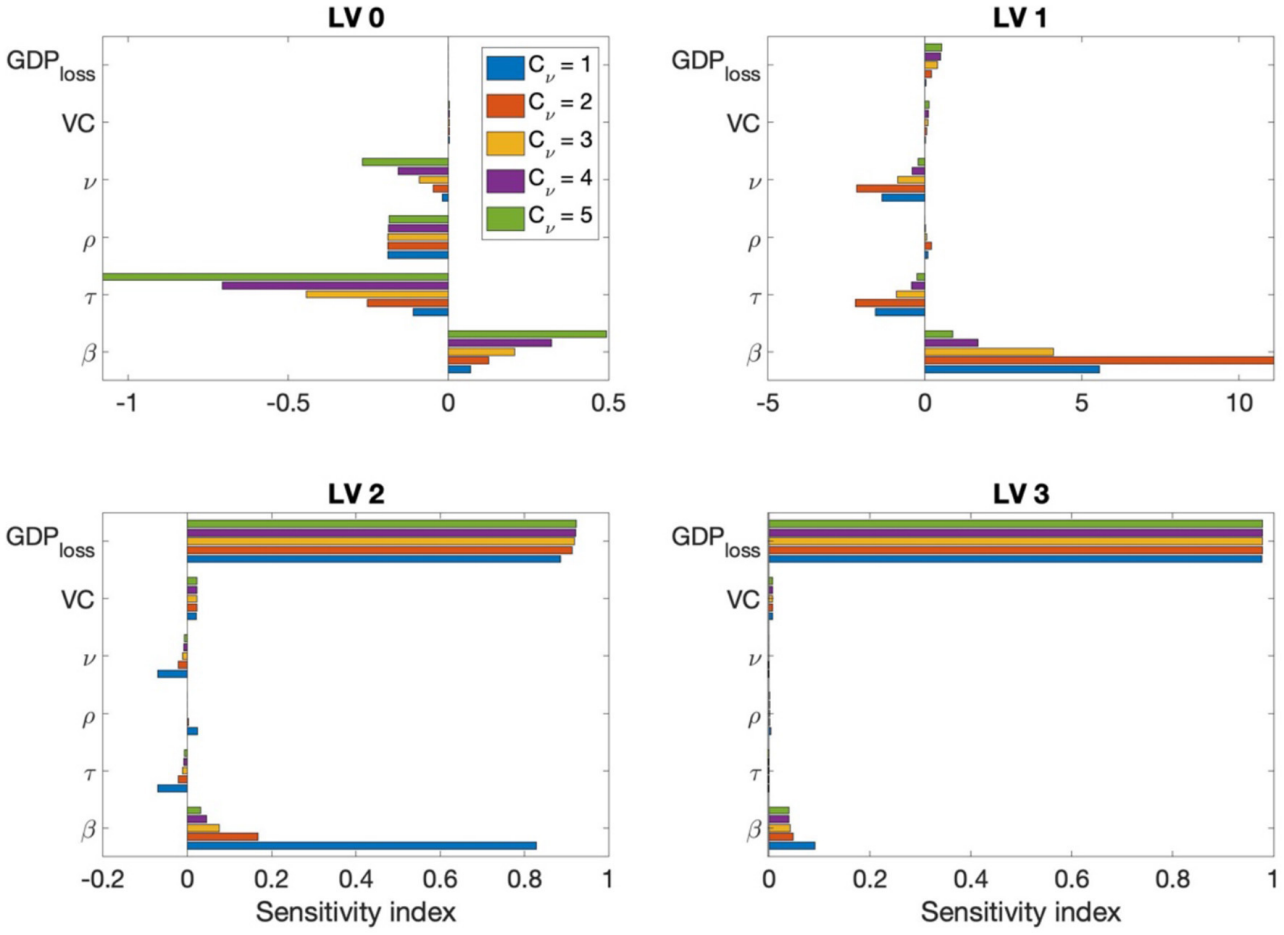
Sensitivity index of β, τ, ρ, ν, the vaccine cost, and GDP loss rate for C_ν_=1, 2, ..., 5 and each SD level.

Sensitivity analyses were conducted to determine the relative importance of the parameters related to cost—that is β, τ, ρ, ν, the vaccine cost, and GDP loss rate—with respect to the disease transmission dynamics. We performed further sensitivity analyses on the model parameters described in Table 1. We used the normalized forward sensitivity index of the total cost (TC) on parameter *p*, defined as T$C_{p}=\frac{\partial \left(TC\right)}{\partial p}\times \frac{p}{TC}$ (33). The TC for one year was computed by varying one parameter by 5% from the baseline value while the rest of the parameters were fixed at their baseline values. For the transmission rate matrix β, all components were increased simultaneously. Figure 7 shows that increases in β, VC, and *GDP*_*loss*_ affected the total cost positively, meaning that when those parameters increased, the total cost also increased; however, an increase in ν_0_ and τ negatively affected the total cost for all cases. *TC*_ρ_ for the probability of unconfirmed asymptomatic cases ρ was either positive or negative, depending on the SD level and rollout speed *C*_ν_. If the number of confirmed cases was high, such as in the case of SD LV 0, a negative value was displayed. This can be understood as, when the number of un-confirmed cases increases, a relatively decreased effect on confirmed cases is seen. However, when the number of confirmed cases is small, an increase in the number of unconfirmed asymptomatic patients leads to an increase in the infection rate in the susceptible population, such that the sensitivity index becomes positive. The change in total cost, according to the change in the transmission rate and vaccine efficacy, was greatest under SD level 1. When the SD level was higher, the change in total cost according to the GDP loss rate was greater.

## Discussion

In this study, we developed an age-structured mathematical model to describe the transmission dynamics of COVID-19 considering vaccination. In the model, we divided the population into eight distinct age groups and estimated the transmission rate for each age group under different levels of social distancing, formulated as an 8 ×8 matrix. Using the model, we investigated the time-dependent dynamics of the number of infected, severe cases, and deaths under varying social distancing levels, vaccine efficiencies, and vaccination rates.

Furthermore, we estimated the total cost of COVID-19, including medical expenses, wage loss, cost due to death, vaccination cost, GDP loss under various vaccine rollout speeds, and vaccine efficiencies under different levels of SD. We found that a faster vaccination rollout speed and higher efficiency resulted in a lower overall cost.

We also investigated the change in the total cost under variation of the rollout speed and disease transmission rate. Figure 4 shows that, when the rollout speed was slow (*C*_ν_=1), SD LV 2 minimized the total cost; otherwise, the total cost was minimized in the case of SD LV1.

Moreover, if the vaccination rate was fast enough (*C*_ν_ ≥ 3), when a sufficient number of vaccines was available, maintaining SD LV1 was the best strategy in terms of both cost minimization and proper medical care of seriously ill patients.

Figure 5 demonstrates that, in order to mitigate the social distancing level, the vaccination coverage rate must be as high as 80%, and the total cost will decrease according to the coverage rate. In particular, when the SD level is alleviated from LV 2 to LV 1, the total cost can be sufficiently reduced even when the coverage rate is about 60%; however, it can be seen that some degree of social distancing must be maintained to prevent further outbreaks.

As it is very important to prevent the collapse of the medical system by reducing the number of seriously ill patients and the number of deaths, it is necessary to devise a control strategy that minimizes damage from a comprehensive perspective, rather than simply planning a strategy from a cost-effective perspective. Figure 3 shows that the rollout speed is important in reducing the number of confirmed cases and serious cases. In the case of SD LV 1, the rollout speed must be as high as *C*_ν_ ≥ 3 in order to sufficiently care for critically ill patients. In case of SD LV 2, even if the rollout speed is *C*_ν_ = 1, severe patients can still receive sufficient medical support.

Omicron variations were not taken into account in the model. Concerning the vaccine efficacy, we assumed two values (0.79 and 0.6) based on the actual efficacy for COVID-19 before Omicron emerged. The results presented in this paper may differ for other efficiency values, but the overall conclusion will not be different.

We assumed the vaccine rollout speed values based on the actual data regarding vaccinations conducted in Korea. In other countries, the vaccination rollout speed may vary more than our hypothesized values.

In this paper, we demonstrated that the vaccination rollout speed is important for both controlling the spread of COVID-19 and reducing costs. In the case of Korea, as the medical infrastructure is solid and the voluntary vaccination rate of the people is high, if the rollout speed of a high-efficiency vaccine is fast enough, economic costs can be reduced by lowering the social distancing level.

## Data availability statement

The original contributions presented in the study are included in the article/Supplementary material, further inquiries can be directed to the corresponding author/s.

## Author contributions

JEK and CHL: conceptualization, funding acquisition, and writing—review & editing. JEK and YC: formal analysis, visualization, and methodology. JEK, HC, and CHL: validation. JEK and HC: writing—original draft. All authors contributed to the article and approved the submitted version.

## Funding

JEK was supported by a National Research Foundation of Korea (NRF) grant and funded by the Korean government (MSIT) (Grant Number NRF-2021R1I1A1A01044426). HC and CHL were supported by a National Research Foundation of Korea (NRF) grant funded by the Korea government (MSIT) (2022R1F1A1064487) and the BK21 Program (Next Generation Education Program for Mathematical Sciences, 4299990414089) funded by the Ministry of Education(MOE, Korea) and National Research Foundation of Korea (NRF).

## Conflict of interest

The authors declare that the research was conducted in the absence of any commercial or financial relationships that could be construed as a potential conflict of interest.

## Publisher's note

All claims expressed in this article are solely those of the authors and do not necessarily represent those of their affiliated organizations, or those of the publisher, the editors and the reviewers. Any product that may be evaluated in this article, or claim that may be made by its manufacturer, is not guaranteed or endorsed by the publisher.
