# Supplementary material for: The economic impact of COVID-19 interventions: A mathematical modeling approach
Source: Front Public Health. 2022 Sep 12;10:993745. doi: 10.3389/fpubh.2022.993745 (PMC9512395; doi:10.3389/fpubh.2022.993745)
Supplement: Supplementary file 1 [file Data_Sheet_1.docx]

Supplementary Material

# Social distancing policies in Korea

As of July 1, 2020, there is a change in the social distancing level standards in Korea. As shown in Table 1, there were from level 1 to 3 before July 1, 2020, thereafter, as Table 2 shows, it is divided into levels 1 to 4. According to Table 1 and Table 2, it can be said that the level 1 and

1.5 of previous social distancing are similar to the level 1 of revised social distancing. In the same way, the level 2 of previous social distancing is similar to the level 2 of revised social distancing, and the level 2.5 and 2.5+*α* of previous social distancing are similar to the level 3 of revised social distancing. During the data fitting period, the social distancing policy is pre-revision and therefore corresponds to LVs 0, 1, 2, 2.5. However, in the paper, LVs 0, 1, 2, and 3 were indicated as standard after revision.

**Table 1.** Social Distancing applied from 7 November 2020 to 30 June 2021

|  | Description | | |
| --- | --- | --- | --- |
| Level | Private gatherings | Events | Assemblies |
| 1 | 500+ people only with advance reporting to local authorities | 500+ people only with advance reporting to local authorities | 500+ people only with advance reporting to local authorities |
| 1.5 | 500+ people only with advance reporting to local authorities | 500+ people only with advance reporting to local authorities | 100+ prohibited |
| 2 | 100+ people prohibited | 100+ people prohibited | 100+ people prohibited |
| 2.5 | 50+ people prohibited | 50+ people prohibited | 50+ people prohibited |
| 2.5+*α* | 5+ people prohibited | 50+ people prohibited | 50+ people prohibited |
| 3 | 10+ people prohibited | 10+ people prohibited | 10+ people prohibited |

**Table 2.** Revised Social Distancing applied after 1 July 2021

|  | Description | | |
| --- | --- | --- | --- |
| Level | Private gatherings | Events | Assemblies |
| 1 | Comply with COVID-19 protocols | 500+ people only with advance reporting to local authorities | 500+ people prohibited |
| 2 | 9+ people prohibited | 100+ people prohibited | 100+ people prohibited |
| 3 | 5+ people prohibited | 50+ people prohibited | 50+ people prohibited |
| 4 | 3+ people prohibited | Events prohibited | Prohibited except 1 person protests |

# Results of parameter fitting for *β*

**Table 3.** The matrices of the transmission rate β for SD level 0, 1, 2, 3

**SD Level 0**

(February 1, 2020 - February 23, 2020; mean($R_{t}$)= 4.663)

| 2.325E-04 | 8.597E-06 | 3.061E-05 | 8.453E-04 | 1.466E-04 | 7.411E-05 | 7.930E-05 | 3.399E-01 |
| --- | --- | --- | --- | --- | --- | --- | --- |
| 5.698E-03 | 1.206E-01 | 2.417E-01 | 1.157E-01 | 3.335E-03 | 1.159E-02 | 3.209E-03 | 1.047E-01 |
| 2.765E-01 | 5.435E-02 | 6.181E-01 | 1.360E-01 | 9.998E-01 | 3.148E-01 | 6.930E-01 | 9.940E-02 |
| 9.485E-04 | 4.675E-04 | 1.348E-04 | 9.269E-01 | 6.655E-04 | 1.953E-03 | 5.378E-04 | 9.889E-01 |
| 9.886E-01 | 5.244E-03 | 4.559E-04 | 2.487E-01 | 7.699E-06 | 1.521E-01 | 8.024E-01 | 1.945E-01 |
| 5.238E-01 | 1.740E-04 | 7.689E-05 | 9.996E-01 | 7.881E-06 | 7.526E-06 | 3.004E-01 | 9.990E-01 |
| 1.234E-02 | 1.818E-03 | 1.352E-06 | 8.415E-01 | 9.028E-05 | 5.909E-04 | 4.865E-03 | 9.994E-01 |
| 1.038E-03 | 5.019E-04 | 5.373E-05 | 2.572E-01 | 2.086E-04 | 2.561E-03 | 6.376E-04 | 9.356E-01 |

**SD Level 1**

(October 12, 2020 - November 23, 2020; mean($R_{t}$)= 1.659)

| 2.325E-04 | 8.597E-06 | 3.061E-05 | 8.453E-04 | 1.466E-04 | 7.411E-05 | 7.930E-05 | 3.399E-01 |
| --- | --- | --- | --- | --- | --- | --- | --- |
| 5.698E-03 | 1.206E-01 | 2.417E-01 | 1.157E-01 | 3.335E-03 | 1.159E-02 | 3.209E-03 | 1.047E-01 |
| 2.765E-01 | 5.435E-02 | 6.181E-01 | 1.360E-01 | 9.998E-01 | 3.148E-01 | 6.930E-01 | 9.940E-02 |
| 9.485E-04 | 4.675E-04 | 1.348E-04 | 9.269E-01 | 6.655E-04 | 1.953E-03 | 5.378E-04 | 9.889E-01 |
| 9.886E-01 | 5.244E-03 | 4.559E-04 | 2.487E-01 | 7.699E-06 | 1.521E-01 | 8.024E-01 | 1.945E-01 |
| 5.238E-01 | 1.740E-04 | 7.689E-05 | 9.996E-01 | 7.881E-06 | 7.526E-06 | 3.004E-01 | 9.990E-01 |
| 1.234E-02 | 1.818E-03 | 1.352E-06 | 8.415E-01 | 9.028E-05 | 5.909E-04 | 4.865E-03 | 9.994E-01 |
| 1.038E-03 | 5.019E-04 | 5.373E-05 | 2.572E-01 | 2.086E-04 | 2.561E-03 | 6.376E-04 | 9.356E-01 |

**SD Level 2**

(November 24, 2020- December 22, 2020; mean($R_{t}$)= 1.201)

| 2.476E-04 | 3.010E-04 | 2.763E-01 | 4.761E-05 | 5.148E-04 | 6.774E-06 | 1.758E-06 | 1.247E-04 |
| --- | --- | --- | --- | --- | --- | --- | --- |
| 5.371E-04 | 1.032E-03 | 3.290E-01 | 4.020E-06 | 3.091E-05 | 3.978E-04 | 6.129E-07 | 2.285E-05 |
| 9.469E-04 | 6.725E-03 | 3.576E-01 | 2.833E-03 | 3.739E-04 | 3.766E-05 | 2.774E-04 | 2.859E-04 |
| 5.000E-03 | 5.271E-03 | 4.233E-01 | 5.783E-03 | 3.086E-03 | 7.617E-06 | 4.314E-03 | 8.406E-03 |
| 1.561E-05 | 3.033E-03 | 3.963E-01 | 2.362E-03 | 3.577E-04 | 1.415E-05 | 2.377E-04 | 2.325E-02 |
| 1.061E-03 | 7.193E-03 | 4.490E-01 | 4.743E-03 | 2.705E-04 | 8.180E-04 | 3.485E-04 | 7.219E-02 |
| 7.510E-05 | 4.682E-05 | 5.933E-01 | 8.982E-06 | 4.977E-05 | 5.844E-05 | 2.655E-05 | 2.197E-05 |
| 3.025E-07 | 1.937E-05 | 6.211E-01 | 8.632E-05 | 2.289E-06 | 3.914E-05 | 9.681E-07 | 1.533E-05 |

**SD Level 3**

(December 22, 2020- February,15,2021; mean($R_{t}$)= 1.005)

| 3.097E-01 | 1.515E-06 | 2.025E-06 | 3.543E-08 | 2.052E-05 | 4.176E-06 | 2.255E-07 | 2.224E-06 |
| --- | --- | --- | --- | --- | --- | --- | --- |
| 2.920E-01 | 2.187E-05 | 4.531E-05 | 1.126E-04 | 9.712E-05 | 7.483E-05 | 5.879E-05 | 2.760E-05 |
| 4.777E-05 | 4.736E-06 | 3.112E-05 | 2.081E-08 | 2.481E-05 | 1.570E-05 | 2.569E-01 | 9.281E-08 |
| 1.239E-03 | 1.450E-01 | 3.526E-05 | 1.839E-05 | 2.284E-05 | 1.281E-05 | 1.565E-01 | 5.076E-06 |
| 2.421E-04 | 1.019E-03 | 7.905E-05 | 1.777E-04 | 1.106E-04 | 9.180E-05 | 2.381E-01 | 5.704E-08 |
| 5.122E-05 | 7.155E-05 | 6.482E-05 | 1.656E-04 | 1.058E-04 | 2.519E-07 | 2.840E-01 | 6.562E-05 |
| 2.416E-01 | 9.972E-02 | 4.611E-04 | 9.365E-04 | 6.897E-04 | 6.158E-04 | 6.388E-03 | 6.069E-04 |
| 1.012E-04 | 9.113E-05 | 3.979E-05 | 4.361E-05 | 1.692E-05 | 2.134E-05 | 2.684E-01 | 2.277E-06 |


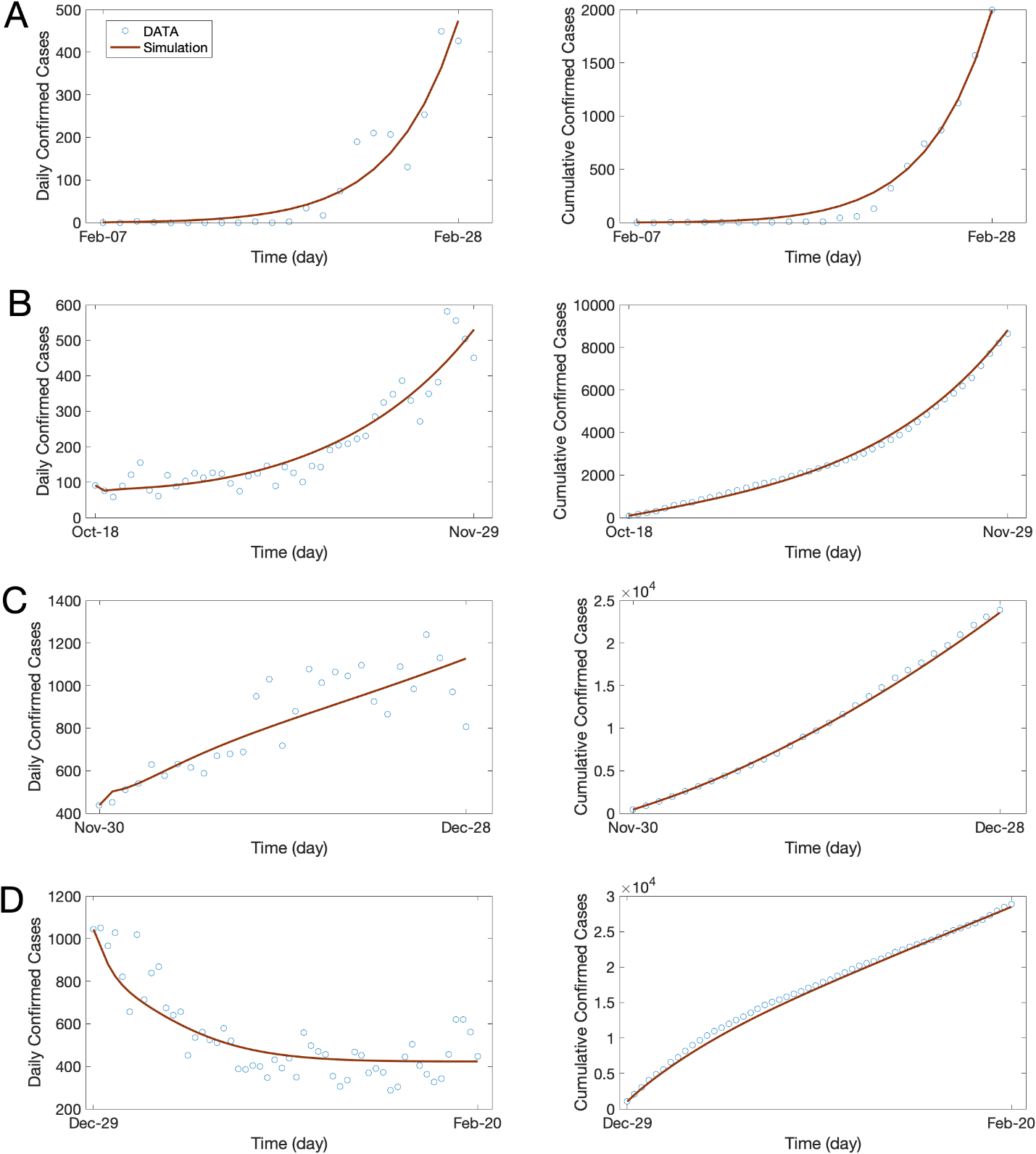


Figure 1. Comparison of the confirmed case data and simulation results for (Left)Daily confirmed cases of all ages (Right) Cumulative confirmed cases of all ages for (A) SD level 0 (B) SD level 1 (C) SD level 2 (D) SD level 3

# The effective reproduction numbers

We computed the effective reproduction number $R_{t}$, which measures the mean number of the secondary cases infected by an infectious individual at time $t$, which is obtained by calculating the spectral radius of the next-generation matrix. Let $x=\left( E_{i},A_{i},I_{i},H_{i}^{M},H_{i}^{H},H_{i}^{I},S_{i},V_{i},R_{i},D_{i} \right)^{T}$ for $i=1, \cdots, 8$. Let $F\left( x \right)$ represents all of the new infections and the net transition rates of the corresponding compartments are represented by $V\left( x \right).$

$$F\left( x \right)=\left( \begin{aligned} (S_{i}+(1-\tau)V_{i})\Lambda_{i} \\ 0 \\ 0 \\ 0 \\ 0 \\ 0 \\ 0 \\ 0 \\ 0 \\ 0 \end{aligned} \right)$$

where $\Lambda_{i}=\sum_{k} \frac{\beta_{ik}(I_{k}+\theta_{k}A_{k})}{N_{k}}.$

$$V\left( x \right)=\left( \begin{aligned} \alpha E_{i} \\ -\rho\alpha E_{i}+\gamma^{A}A_{i} \\ -\left( 1-\rho\right)\rho E_{i}+qI_{i} \\ -{q\delta}^{M}I_{i}+\gamma^{M}H_{i}^{M} \\ -q\delta^{H}I_{i}+\gamma^{H}H_{i}^{H} \\ -q\delta^{I}I_{i}+\gamma^{I}H_{i}^{I} \\ \Lambda_{i}S_{i}+\phi_{i}\upsilon\\ -\phi_{i}\upsilon+(1-\tau)\Lambda_{i}V_{i} \\ {-\gamma}^{A}A_{i}-\gamma^{M}H_{i}^{M}-\gamma^{H}H_{i}^{H}-\left( 1-\kappa^{I} \right)\gamma^{I}H_{i}^{I} \\ {-\kappa}^{I}\eta^{I}H_{i}^{I} \end{aligned} \right)$$

Then, $\mathbf{F}$ and $\mathbf{V}$ are $48*48$ matrices given by $\mathbf{F}=\left[ \frac{\partial F_{i}}{\partial x_{j}}(x_{0}) \right]$ and $\mathbf{V}=\left[ \frac{\partial V_{i}}{\partial x_{j}}(x_{0}) \right]$ with $1\leq i,j\leq48$ as a part of next generation operator where $x_{0}$ is the disease-free state. Thus, we obtain

$$\mathbf{F}\boldsymbol{=}\left[ \begin{matrix} 0_{8,8} & \theta_{A}M_{A} & M_{A} & 0_{8,8} & 0_{8,8} & 0_{8,8} \\ 0_{8,8} & 0_{8,8} & 0_{8,8} & 0_{8,8} & 0_{8,8} & 0_{8,8} \\ 0_{8,8} & 0_{8,8} & 0_{8,8} & 0_{8,8} & 0_{8,8} & 0_{8,8} \\ 0_{8,8} & 0_{8,8} & 0_{8,8} & 0_{8,8} & 0_{8,8} & 0_{8,8} \\ 0_{8,8} & 0_{8,8} & 0_{8,8} & 0_{8,8} & 0_{8,8} & 0_{8,8} \\ 0_{8,8} & 0_{8,8} & 0_{8,8} & 0_{8,8} & 0_{8,8} & 0_{8,8} \end{matrix} \right].$$

Here $M_{A}$ is the matrix computed as

$$M_{A}=diag\{\left( S_{1}+\left( 1-\tau\right)V_{1} \right),\left( S_{2}+\left( 1-\tau\right)V_{2} \right), \cdots,\left( S_{8}+\left( 1-\tau\right)V_{8} \right){\}}_{8}*B*diag\{\frac{1}{N_{1}},\frac{1}{N_{2}},\cdots,\frac{1}{N_{8}}{\}}_{8},$$

where $B=[\beta_{ij}]$, $S_{i}$ is the susceptible population of age group $i$, $V_{i}$ is the vaccinated population of age group $i$, $diag\{{\}}_{n}$ denotes the diagonal matrix with $n$ diagonal entries.

$$\mathbf{V}\boldsymbol{=}\left[ \begin{matrix} M_{B} & 0_{8,8} & 0_{8,8} & 0_{8,8} & 0_{8,8} & 0_{8,8} \\ -\rho M_{B} & M_{C} & 0_{8,8} & 0_{8,8} & 0_{8,8} & 0_{8,8} \\ -(1-\rho{)M}_{B} & 0_{8,8} & M_{D} & 0_{8,8} & 0_{8,8} & 0_{8,8} \\ 0_{8,8} & 0_{8,8} & {-\delta}^{M}M_{D} & M_{E} & 0_{8,8} & 0_{8,8} \\ 0_{8,8} & 0_{8,8} & {-\delta}^{H}M_{D} & 0_{8,8} & M_{F} & 0_{8,8} \\ 0_{8,8} & 0_{8,8} & {-\delta}^{I}M_{D} & 0_{8,8} & 0_{8,8} & M_{G} \end{matrix} \right]$$

where $M_{B}=\alpha*\mathbf{I}_{8}$,$M_{C}=\gamma^{A}*\mathbf{I}_{8}$, $M_{D}=q*\mathbf{I}_{8}$,$M_{E}=\gamma^{M}*\mathbf{I}_{8}$, $M_{F}=\gamma^{H}*\mathbf{I}_{8}$, $M_{G}=\gamma^{I}*\mathbf{I}_{8}$, and $\mathbf{I}_{8}$ is the size 8 identity matrix.

Then, the inverse matrix of $\mathbf{V}$ is

$$\mathbf{V}^{\boldsymbol{-1}}\boldsymbol{=}\left[ \begin{matrix} {M_{B}}^{-1} & 0_{8,8} & 0_{8,8} & 0_{8,8} & 0_{8,8} & 0_{8,8} \\ \rho{M_{C}}^{-1} & {M_{C}}^{-1} & 0_{8,8} & 0_{8,8} & 0_{8,8} & 0_{8,8} \\ (1-\rho){M_{D}}^{-1} & 0_{8,8} & {M_{D}}^{-1} & 0_{8,8} & 0_{8,8} & 0_{8,8} \\ (1-\rho){{\delta^{M}M}_{E}}^{-1} & 0_{8,8} & \delta^{M}{M_{E}}^{-1} & {M_{E}}^{-1} & 0_{8,8} & 0_{8,8} \\ (1-\rho){{\delta^{H}M}_{F}}^{-1} & 0_{8,8} & \delta^{H}{M_{F}}^{-1} & 0_{8,8} & {M_{F}}^{-1} & 0_{8,8} \\ (1-\rho)\delta^{I}{M_{G}}^{-1} & 0_{8,8} & \delta^{I}{M_{G}}^{-1} & 0_{8,8} & 0_{8,8} & {M_{G}}^{-1} \end{matrix} \right]$$

.

Hence, one can obtain the next generation matrix $\mathbf{G}$ as

$\mathbf{G}\boldsymbol{=}\mathbf{F}\mathbf{V}^{\boldsymbol{-1}}\boldsymbol{=}\left[ \begin{matrix} (\frac{\rho\theta_{A}}{\gamma^{A}}+\frac{1-\rho}{q})M_{A} & \frac{\theta_{A}}{\gamma^{A}}M_{A} & \frac{1}{q}M_{A} & 0_{8,8} & 0_{8,8} & 0_{8,8} \\ 0_{8,8} & 0_{8,8} & 0_{8,8} & 0_{8,8} & 0_{8,8} & 0_{8,8} \\ 0_{8,8} & 0_{8,8} & 0_{8,8} & 0_{8,8} & 0_{8,8} & 0_{8,8} \\ 0_{8,8} & 0_{8,8} & 0_{8,8} & 0_{8,8} & 0_{8,8} & 0_{8,8} \\ 0_{8,8} & 0_{8,8} & 0_{8,8} & 0_{8,8} & 0_{8,8} & 0_{8,8} \\ 0_{8,8} & 0_{8,8} & 0_{8,8} & 0_{8,8} & 0_{8,8} & 0_{8,8} \end{matrix} \right]$.

Finally, the effective reproduction number $R_{t}$ is computed as the spectral radius $\rho(\mathbf{G})$ of the next generation matrix $\mathbf{G}$, i.e., $R_{t}=\rho(\mathbf{G})$.

$R_{t}=\rho\left( \mathbf{G} \right)=(\frac{\rho\theta_{A}}{\gamma^{A}}+\frac{1-\rho}{q})\rho\left( \boldsymbol{M}_{\boldsymbol{A}} \right)$.

# The effect of rollout speed of vaccination on disease transmission

**Table 4.** The numbers of cumulative confirmed cases, cumulative death, and maximum hospitalized population in ICU for various social distancing level, rollout speed (*C_v_*), and vaccine efficacy ($\tau).$Bold text indicates when the maximum number of inpatients with severe symptoms is less than the ICU bed capacity.

|  |  | $\tau=0.79$ | | | $\tau=0.6$ | | |
| --- | --- | --- | --- | --- | --- | --- | --- |
| SD | *C_v_* | Cumulative cases | Cumulative death | Max($H^{I}$) | Cumulative cases | Cumulative death | Max($H^{I}$) |
| LV 0 | 1 | 4.177E+7 | 4.026E+5 | 5.808E+5 | 4.228E+7 | 4.105E+5 | 5.957E+5 |
|  | 2 | 4.086E+7 | 3.884E+5 | 5.459E+5 | 4.199E+7 | 4.061E+5 | 5.778E+5 |
|  | 3 | 3.977E+7 | 3.712E+5 | 5.071E+5 | 4.168E+7 | 4.012E+5 | 5.584E+5 |
|  | 4 | 3.844E+7 | 3.501E+5 | 4.636E+5 | 4.134E+7 | 3.956E+5 | 5.373E+5 |
|  | 5 | 3.676E+7 | 3.240E+5 | 4.146E+5 | 4.097E+7 | 3.893E+5 | 5.144E+5 |
| LV 1 | 1 | 1.463E+7 | 1.185E+5 | 6.828E+4 | 2.060E+7 | 1.715E+5 | 1.059E+5 |
|  | 2 | 1.981E+6 | 1.509E+4 | 8.657E+3 | 5.706E+6 | 4.412E+4 | 2.312E+4 |
|  | 3 | 5.021E+5 | 3,799.9 | **2,535.4** | 1.361E+6 | 10,338. | 5,652.1 |
|  | 4 | 2.322E+5 | 1,757.5 | **1,311.5** | 5.626E+5 | 4,266.4 | **2,459.2** |
|  | 5 | 1.418E+5 | 1,075.1 | **872.2** | 3.200E+5 | 2,427.7 | **1,461** |
| LV 2 | 1 | 2.729E+5 | 3,310.3 | **1,151.8** | 4.334E+5 | 5,249.6 | **1,572.1** |
|  | 2 | 1.087E+5 | 1,309.2 | **674.1** | 1.514E+5 | 1,830.8 | **807.9** |
|  | 3 | 7.094E+4 | 848.6 | **540.2** | 9.442E+4 | 1,135.3 | **624.9** |
|  | 4 | 5.424E+4 | 644.7 | **470.7** | 7.069E+4 | 845.6 | **536.8** |
|  | 5 | 4.476E+4 | 529. | **425.8** | 5.774E+4 | 687.4 | **482.1** |
| LV 3 | 1 | 6.876E+4 | 587.5 | **153.9** | 7.787E+4 | 675.4 | **155.1** |
|  | 2 | 4.825E+4 | 379.6 | **149.8** | 5.930E+4 | 484.3 | **151.6** |
|  | 3 | 4.154E+4 | 309.2 | **146.7** | 5.320E+4 | 418.9 | **148.9** |
|  | 4 | 3.825E+4 | 274.6 | **144.** | 5.022E+4 | 386.9 | **146.5** |
|  | 5 | 3.628E+4 | 253.9 | **141.7** | 4.844E+4 | 367.7 | **144.5** |

# Cost estimation

**Table 5.** Cost values for the case of admission to the treatment center for mild patients

| SD | *C_v_* | Medical expenses | Wage loss | Death | Vaccination | GDP loss | Total |
| --- | --- | --- | --- | --- | --- | --- | --- |
| LV 0 | 1 | 1.877E+11 | 4.335E+10 | 5.476E+9 | 2.176E+9 | 0 | 2.387E+11 |
|  | 2 | 1.836E+11 | 4.257E+10 | 5.323E+9 | 2.176E+9 | 0 | 2.337E+11 |
|  | 3 | 1.785E+11 | 4.163E+10 | 5.135E+9 | 2.176E+9 | 0 | 2.275E+11 |
|  | 4 | 1.723E+11 | 4.045E+10 | 4.901E+9 | 2.176E+9 | 0 | 2.198E+11 |
|  | 5 | 1.643E+11 | 3.893E+10 | 4.605E+9 | 2.176E+9 | 0 | 2.100E+11 |
| LV 1 | 1 | 6.344E+10 | 1.532E+10 | 1.688E+9 | 2.176E+9 | 3.290E+9 | 8.592E+10 |
|  | 2 | 8.484E+9 | 2.077E+9 | 2.193E+8 | 2.176E+9 | 3.290E+9 | 1.625E+10 |
|  | 3 | 2.148E+9 | 5.265E+8 | 5.535E+7 | 2.176E+9 | 3.290E+9 | 8.196E+9 |
|  | 4 | 9.931E+8 | 2.434E+8 | 2.560E+7 | 2.176E+9 | 3.290E+9 | 6.728E+9 |
|  | 5 | 6.065E+8 | 1.486E+8 | 1.565E+7 | 2.176E+9 | 3.290E+9 | 6.237E+9 |
| LV 2 | 1 | 1.322E+9 | 2.827E+8 | 4.390E+7 | 2.176E+9 | 2.961E+10 | 3.344E+10 |
|  | 2 | 5.250E+8 | 1.125E+8 | 1.738E+7 | 2.176E+9 | 2.961E+10 | 3.244E+10 |
|  | 3 | 3.418E+8 | 7.337E+7 | 1.128E+7 | 2.176E+9 | 2.961E+10 | 3.222E+10 |
|  | 4 | 2.607E+8 | 5.607E+7 | 8.581E+6 | 2.176E+9 | 2.961E+10 | 3.211E+10 |
|  | 5 | 2.146E+8 | 4.625E+7 | 7.048E+6 | 2.176E+9 | 2.961E+10 | 3.206E+10 |
| LV 3 | 1 | 2.938E+8 | 6.807E+7 | 8.423E+6 | 2.176E+9 | 1.053E+11 | 1.078E+11 |
|  | 2 | 1.981E+8 | 4.655E+7 | 5.554E+6 | 2.176E+9 | 1.053E+11 | 1.077E+11 |
|  | 3 | 1.668E+8 | 3.952E+7 | 4.584E+6 | 2.176E+9 | 1.053E+11 | 1.077E+11 |
|  | 4 | 1.514E+8 | 3.606E+7 | 4.107E+6 | 2.176E+9 | 1.053E+11 | 1.077E+11 |
|  | 5 | 1.423E+8 | 3.399E+7 | 3.823E+6 | 2.176E+9 | 1.053E+11 | 1.077E+11 |

**Table 6.** Cost values for the case of home treatment for mild patients

| SD | *C_v_* | Medical expenses | Wage loss | Death | Vaccination | GDP loss | Total |
| --- | --- | --- | --- | --- | --- | --- | --- |
| LV 0 | 1 | 1.173E+11 | 2.884E+10 | 5.476E+9 | 2.176E+9 | 0 | 1.538E+11 |
|  | 2 | 1.148E+11 | 2.836E+10 | 5.323E+9 | 2.176E+9 | 0 | 1.507E+11 |
|  | 3 | 1.117E+11 | 2.776E+10 | 5.135E+9 | 2.176E+9 | 0 | 1.468E+11 |
|  | 4 | 1.077E+11 | 2.701E+10 | 4.901E+9 | 2.176E+9 | 0 | 1.418E+11 |
|  | 5 | 1.027E+11 | 2.603E+10 | 4.605E+9 | 2.176E+9 | 0 | 1.355E+11 |
| LV 1 | 1 | 3.849E+10 | 1.015E+10 | 1.688E+9 | 2.176E+9 | 3.290E+9 | 5.579E+10 |
|  | 2 | 5.088E+9 | 1.372E+9 | 2.193E+8 | 2.176E+9 | 3.290E+9 | 1.215E+10 |
|  | 3 | 1.286E+9 | 3.478E+8 | 5.535E+7 | 2.176E+9 | 3.290E+9 | 7.155E+9 |
|  | 4 | 5.946E+8 | 1.608E+8 | 2.560E+7 | 2.176E+9 | 3.290E+9 | 6.247E+9 |
|  | 5 | 3.632E+8 | 9.816E+7 | 1.565E+7 | 2.176E+9 | 3.290E+9 | 5.943E+9 |
| LV 2 | 1 | 8.802E+8 | 1.924E+8 | 4.390E+7 | 2.176E+9 | 2.961E+10 | 3.291E+10 |
|  | 2 | 3.486E+8 | 7.649E+7 | 1.738E+7 | 2.176E+9 | 2.961E+10 | 3.223E+10 |
|  | 3 | 2.264E+8 | 4.985E+7 | 1.128E+7 | 2.176E+9 | 2.961E+10 | 3.208E+10 |
|  | 4 | 1.723E+8 | 3.806E+7 | 8.581E+6 | 2.176E+9 | 2.961E+10 | 3.201E+10 |
|  | 5 | 1.416E+8 | 3.137E+7 | 7.048E+6 | 2.176E+9 | 2.961E+10 | 3.197E+10 |
| LV 3 | 1 | 1.739E+8 | 4.441E+7 | 8.423E+6 | 2.176E+9 | 1.053E+11 | 1.077E+11 |
|  | 2 | 1.120E+8 | 2.991E+7 | 5.554E+6 | 2.176E+9 | 1.053E+11 | 1.076E+11 |
|  | 3 | 9.175E+7 | 2.517E+7 | 4.584E+6 | 2.176E+9 | 1.053E+11 | 1.076E+11 |
|  | 4 | 8.179E+7 | 2.284E+7 | 4.107E+6 | 2.176E+9 | 1.053E+11 | 1.076E+11 |
|  | 5 | 7.584E+7 | 2.145E+7 | 3.823E+6 | 2.176E+9 | 1.053E+11 | 1.076E+11 |

# The effects of SD level mitigation


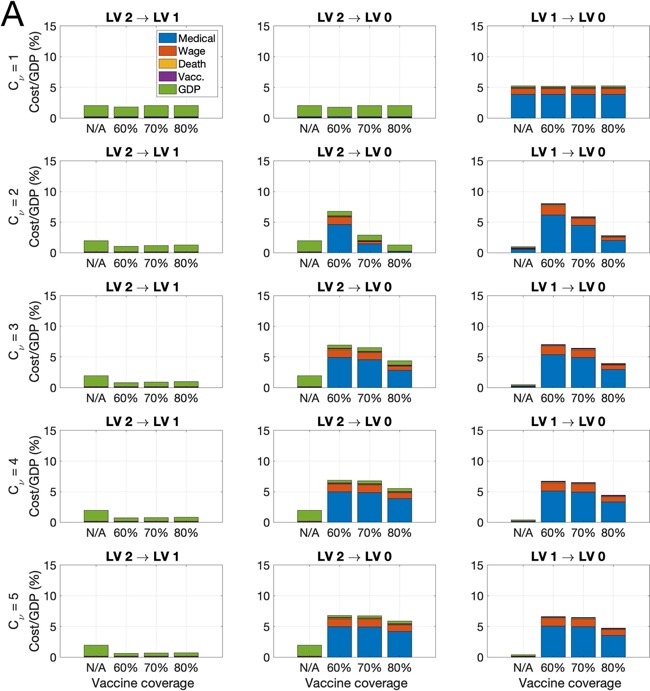

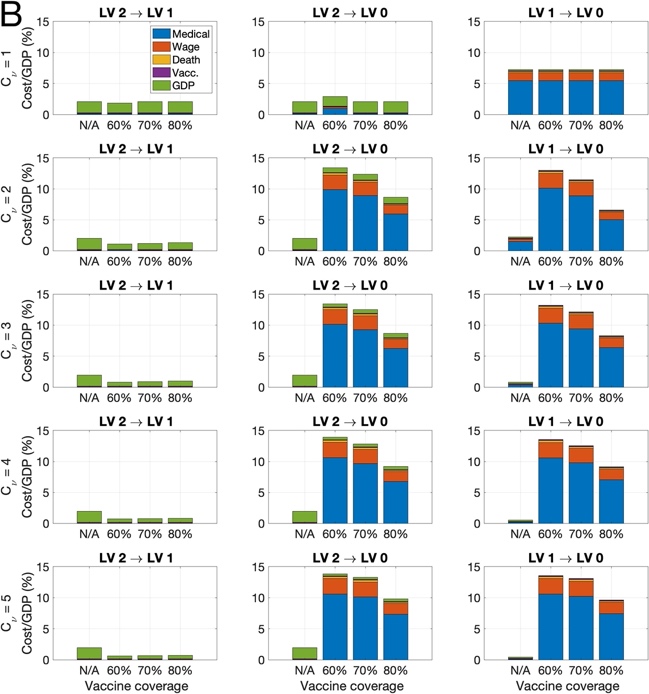


Figure 2. Ratio of cost to GPD for each SD level mitigation scenario when the vaccination coverage rate is reached at 60, 70, 80% (N/A indicates that social distancing easing is not implemented.) (Top) Admission to the treatment center for mild patients (Bottom) Home treatment for mild patients for $\boldsymbol{(A) \tau=0.79}$ and (B) $\boldsymbol{\tau=0.6}$ in case of $\boldsymbol{C}_{\boldsymbol{\nu}}\boldsymbol{=1,2,...,5}$.


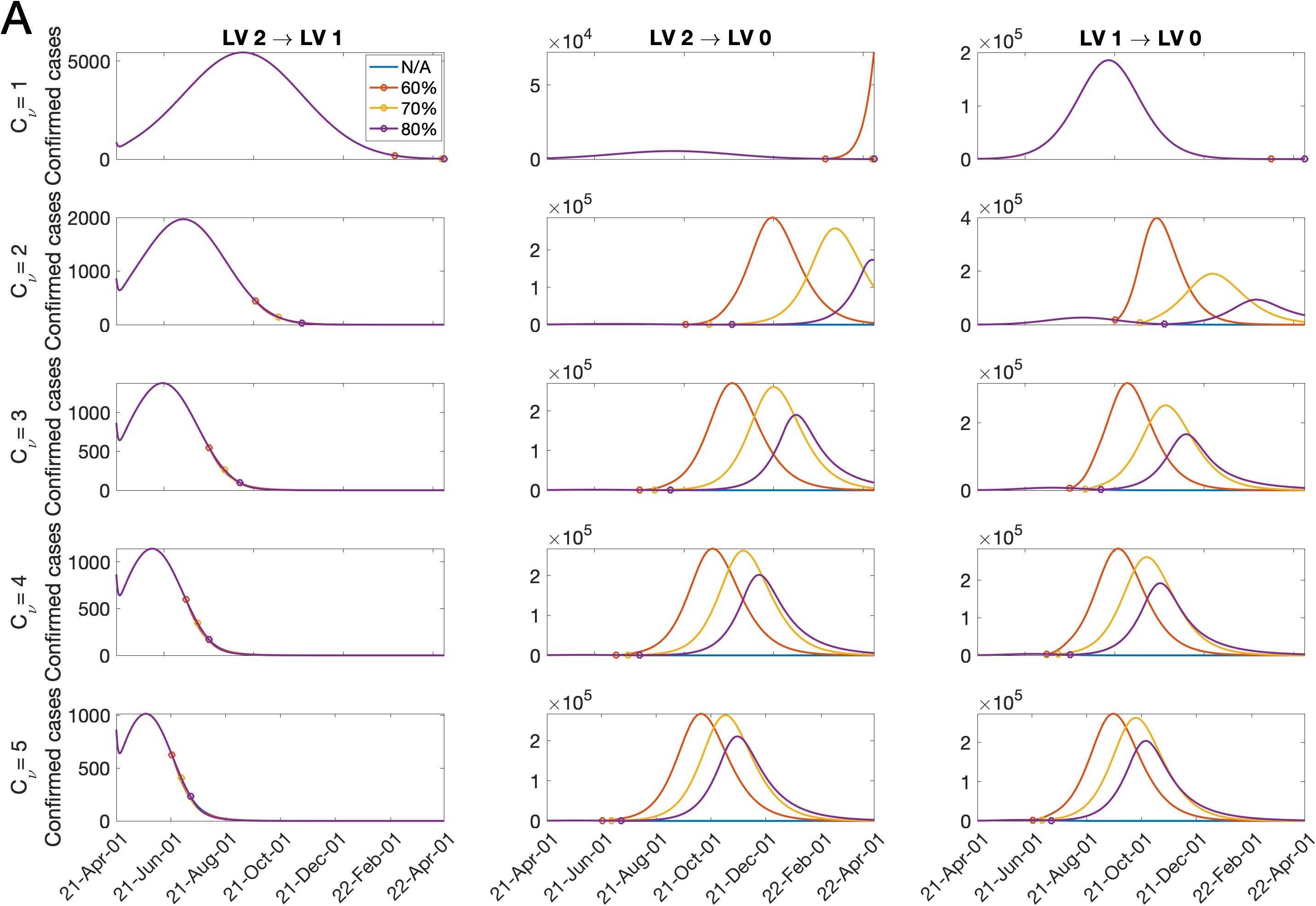


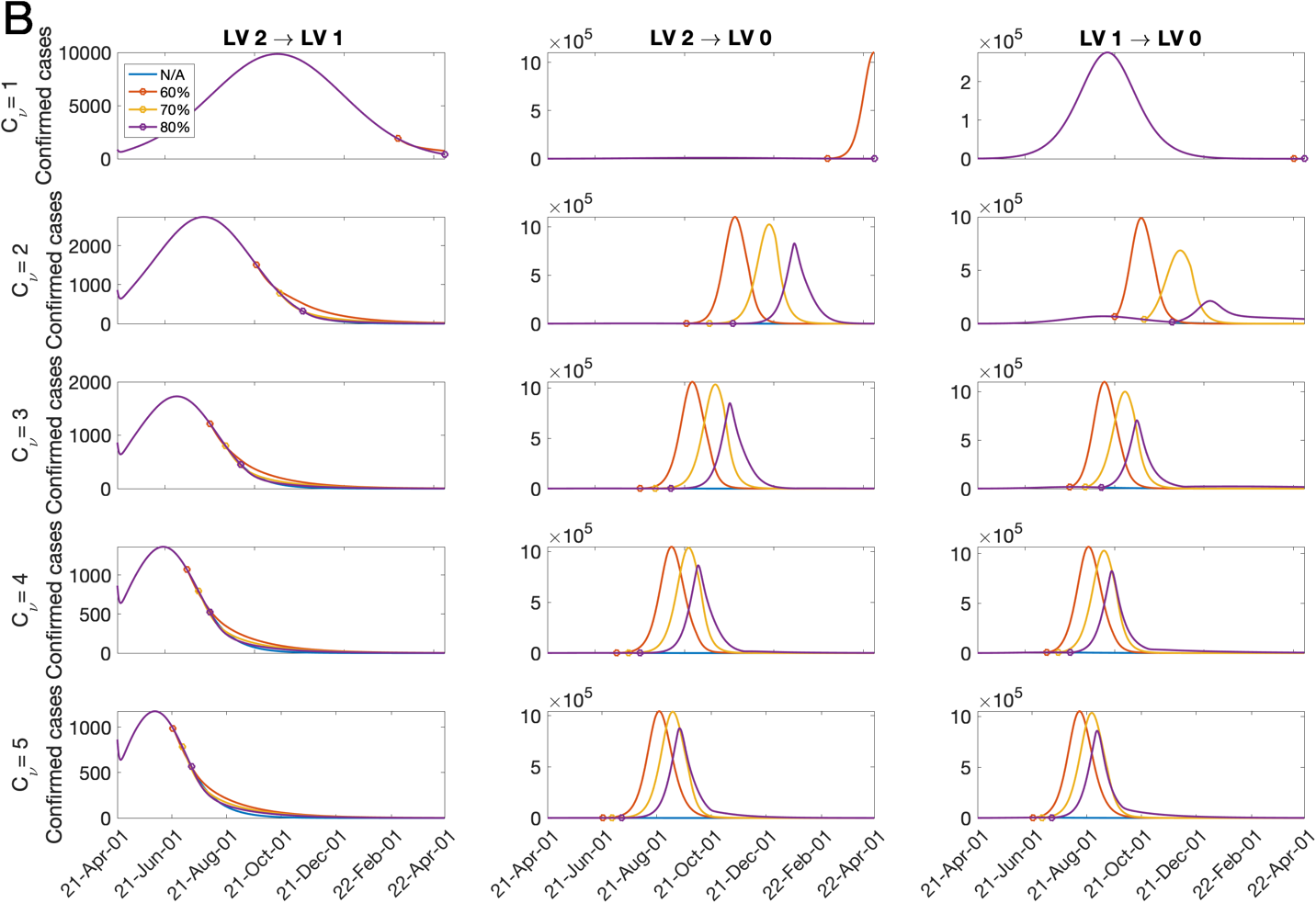


Figure 3. Daily confirmed cases for each SD level mitigation scenario when the vaccination coverage rate is reached at 60, 70, 80% for $\boldsymbol{C}_{\boldsymbol{\nu}}\boldsymbol{=1,2,..., 5}$ for $\boldsymbol{(A) \tau=0.79}$ and (b) $\boldsymbol{\tau=0.6}$.


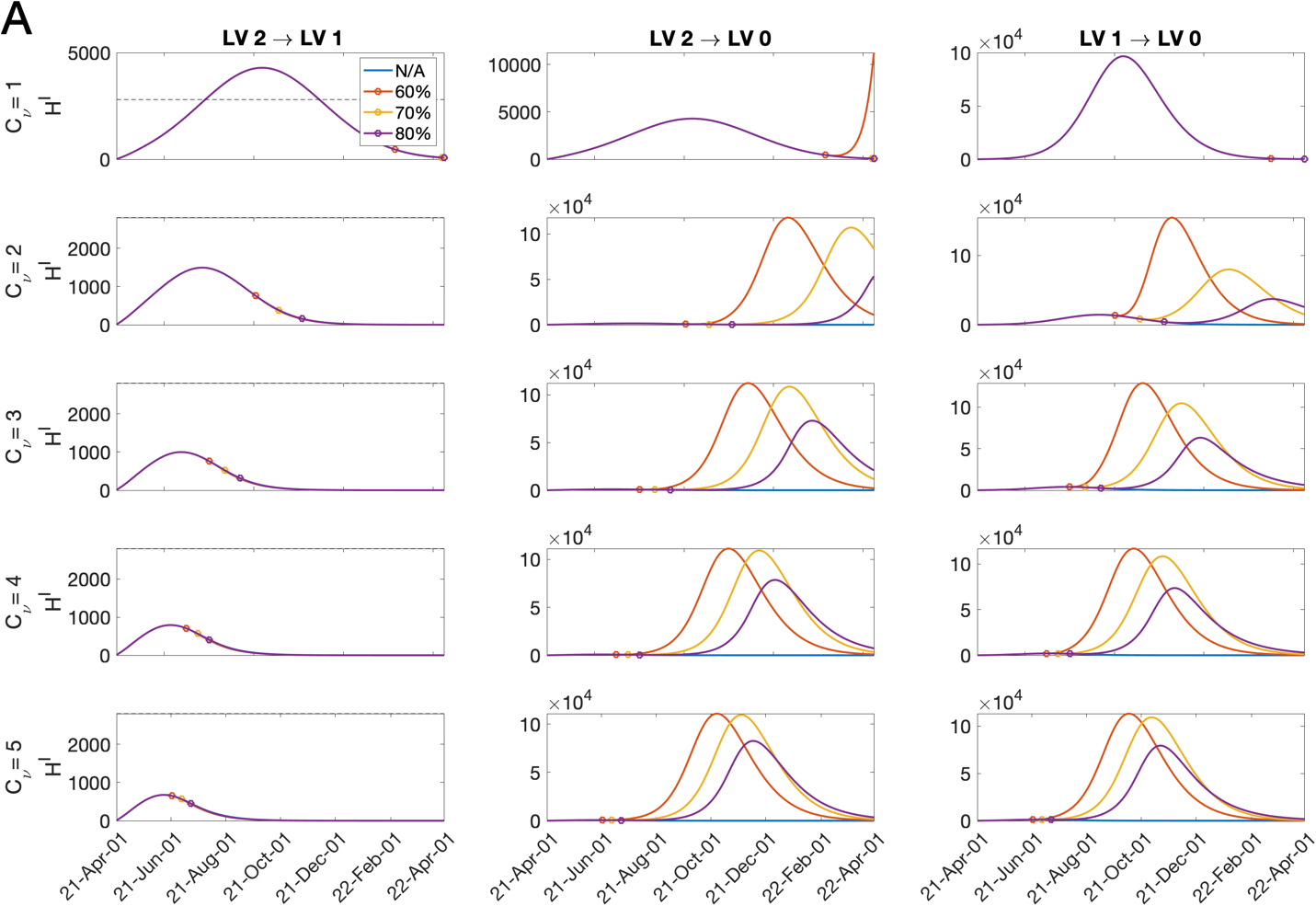


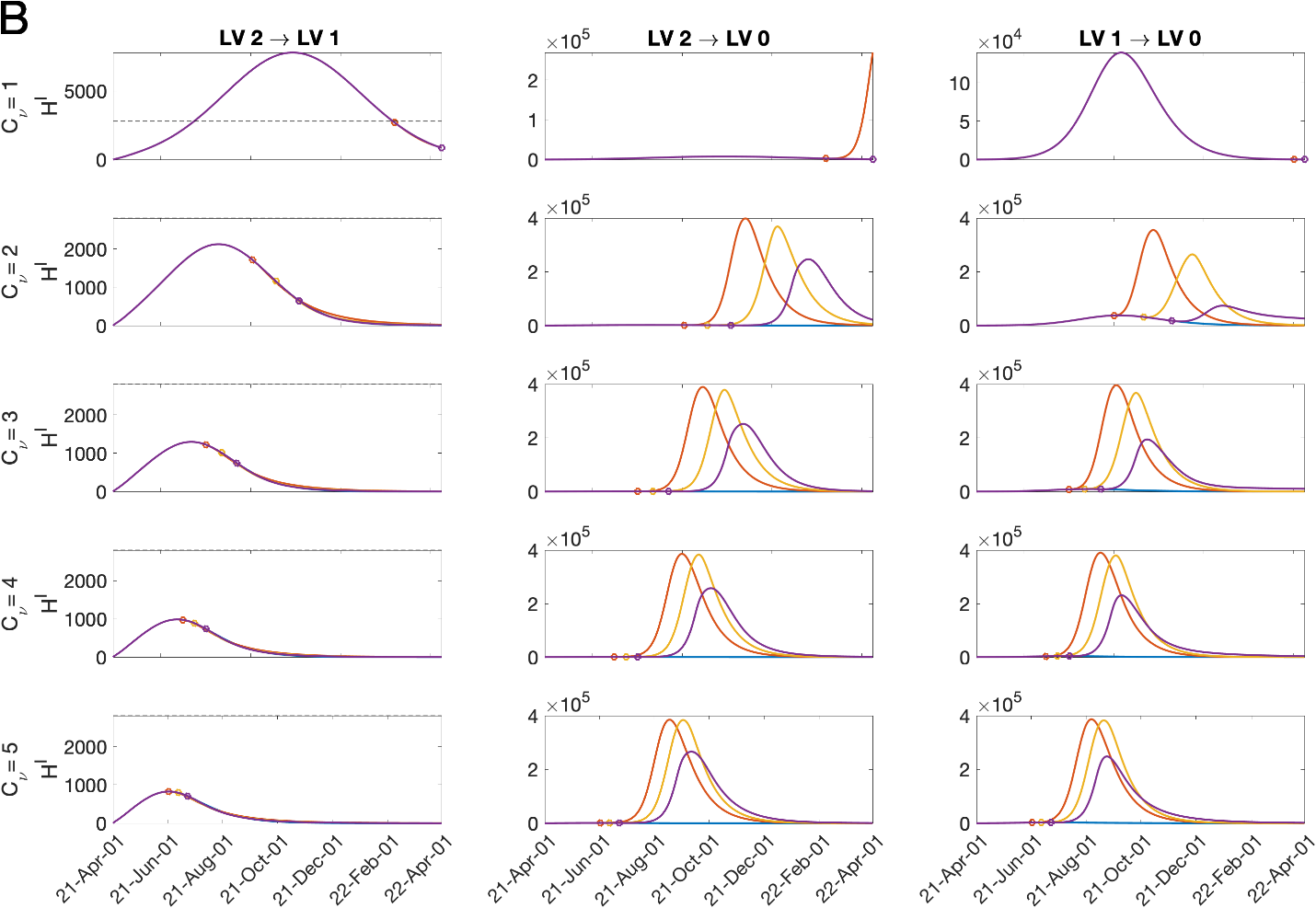


Figure 4. Hospitalized population with severe symptoms for each SD level mitigation scenario when the vaccination coverage rate is reached at 60, 70, 80% for $\boldsymbol{C}_{\boldsymbol{\nu}}\boldsymbol{=1,2,..., 5}$ for $\boldsymbol{(A) \tau=0.79}$ and (b) $\boldsymbol{\tau=0.6}$. The dashed line represents the number of ICU bed for COVID-19 patients in Korea.


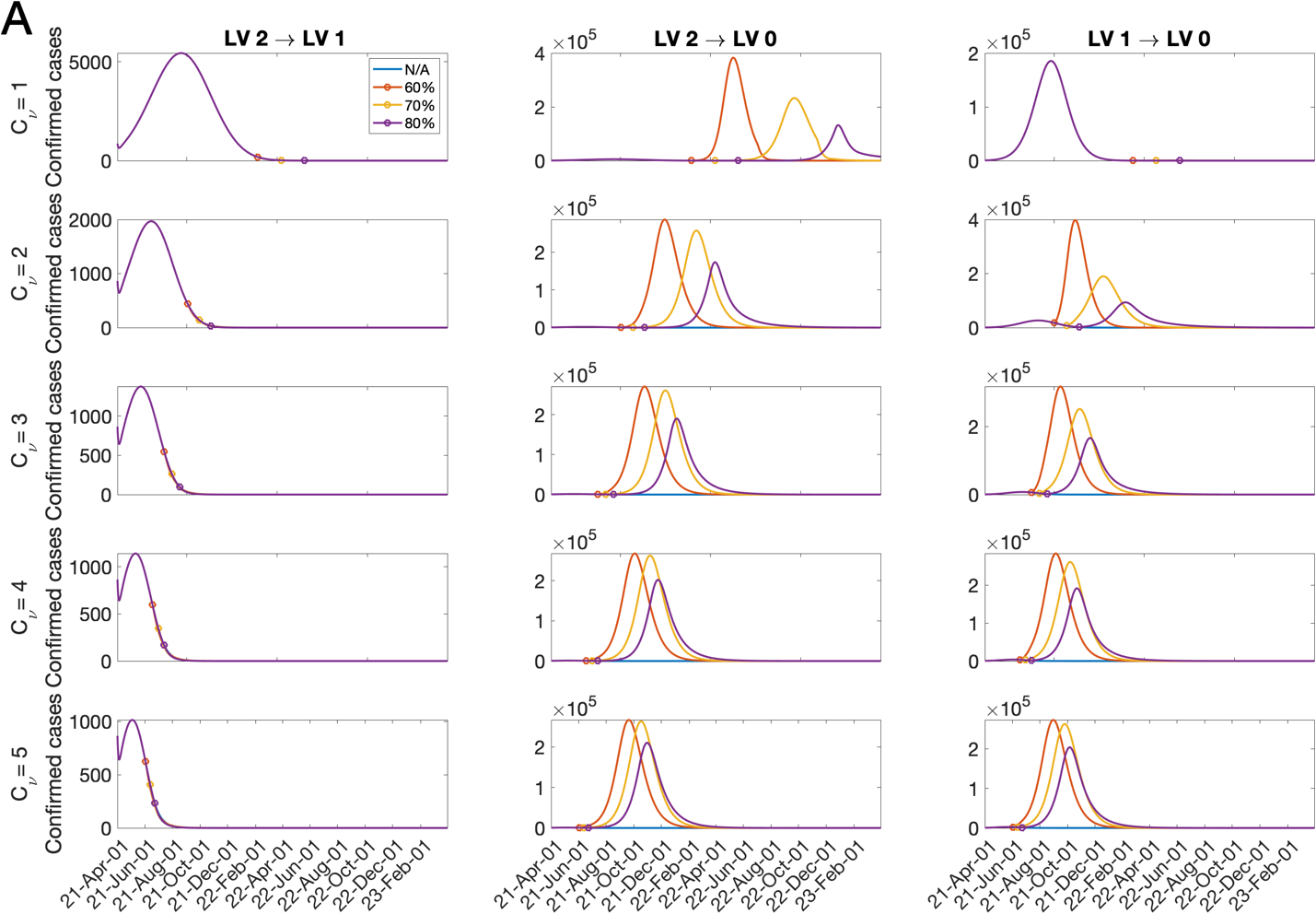


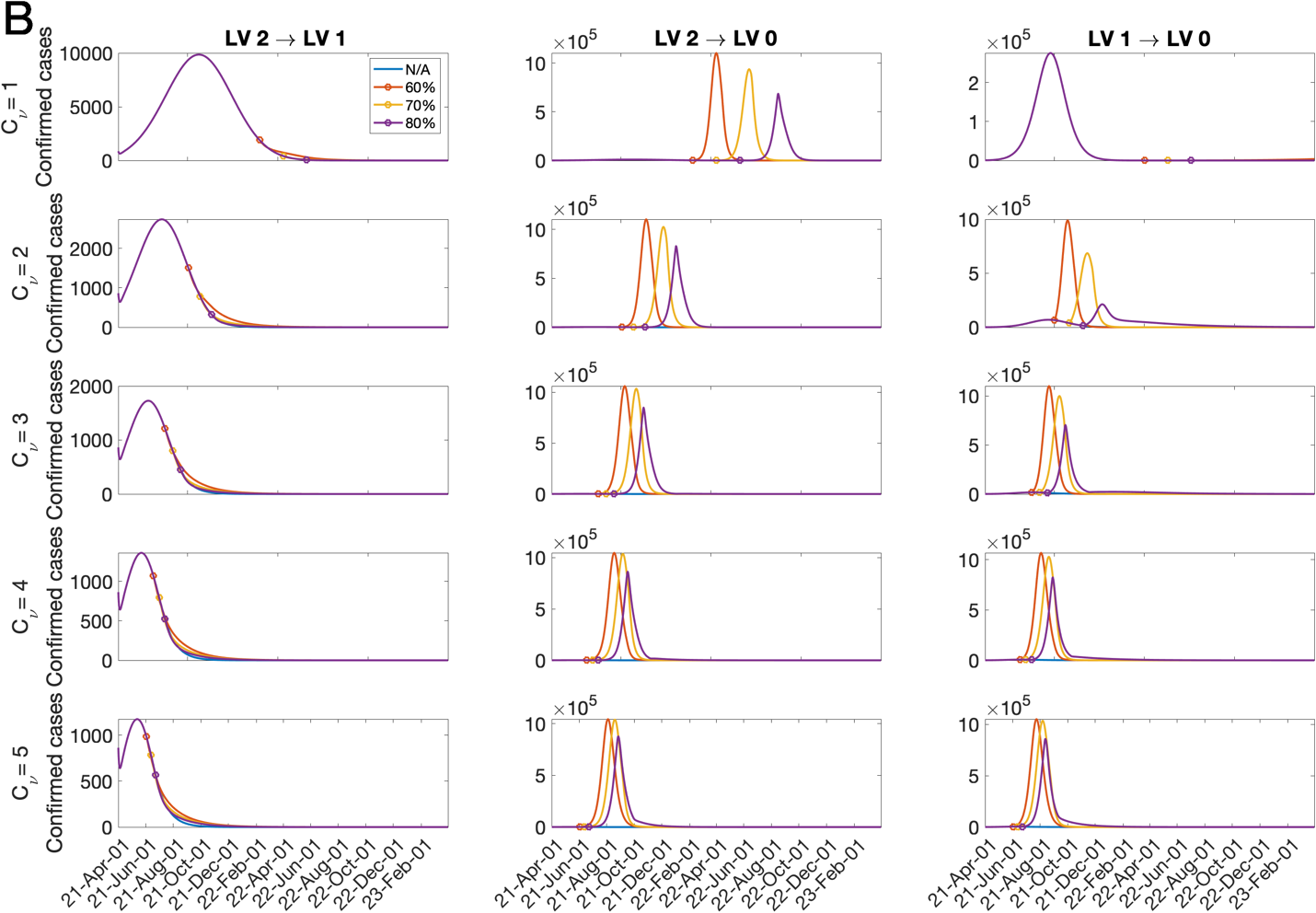


Figure 5. Daily confirmed cases for each SD level mitigation scenario when the vaccination coverage rate is reached at 60, 70, 80% for $\boldsymbol{C}_{\boldsymbol{\nu}}\boldsymbol{=1,2,..., 5}$ for $\boldsymbol{(A) \tau=0.79}$ and (B) $\boldsymbol{\tau=0.6}$. Simulation duration is 2 years.

**Table 7.** Cumulative confirmed cases for each SD level mitigation scenario when the vaccination coverage rate is reached at 60, 70, 80% for $C_{\nu}=3, 4, 5$ for $\tau=0.79$and $0.6$ Simulation duration is (A) 1 year and (B) 2 years .

| (A) |  | SD LV 2 → LV 1 | | | | SD LV 2 → LV 0 | | | | SD LV 1 → LV 0 | | | |
| --- | --- | --- | --- | --- | --- | --- | --- | --- | --- | --- | --- | --- | --- |
| $\tau$ | $C_{\nu}$ | N/A | 60% | 70% | 80% | N/A | 60% | 70% | 80% | N/A | 60% | 70% | 80% |
| 0.79 | $1$ | 8.840E+5 | 8.839E+5 | 8.840E+5 | 8.840E+5 | 8.840E+5 | 1.662E+6 | 8.840E+5 | 8.840E+5 | 1.690E+7 | 1.690E+7 | 1.690E+7 | 1.690E+7 |
|  | $2$ | 2.191E+5 | 2.190E+5 | 2.190E+5 | 2.191E+5 | 2.191E+5 | 2.076E+7 | 1.754E+7 | 5.235E+6 | 2.635E+6 | 2.402E+7 | 1.835E+7 | 9.603E+6 |
|  | $3$ | 1.205E+5 | 1.201E+5 | 1.201E+5 | 1.203E+5 | 1.205E+5 | 2.029E+7 | 1.993E+7 | 1.284E+7 | 6.363E+5 | 2.155E+7 | 1.988E+7 | 1.238E+7 |
|  | 4 | 8.416E+4 | 8.364E+4 | 8.372E+4 | 8.392E+4 | 8.416E+4 | 2.021E+7 | 2.005E+7 | 1.441E+7 | 2.831E+5 | 2.063E+7 | 2.005E+7 | 1.375E+7 |
|  | 5 | 6.565E+4 | 6.504E+4 | 6.512E+4 | 6.531E+4 | 6.565E+4 | 2.019E+7 | 2.009E+7 | 1.527E+7 | 1.687E+5 | 2.036E+7 | 2.009E+7 | 1.468E+7 |
| 0.6 | $1$ | 1.808E+6 | 1.813E+6 | 1.808E+6 | 1.808E+6 | 1.808E+6 | 1.869E+7 | 1.808E+6 | 1.808E+6 | 2.276E+7 | 2.276E+7 | 2.276E+7 | 2.276E+7 |
|  | $2$ | 3.583E+5 | 3.776E+5 | 3.630E+5 | 3.595E+5 | 3.583E+5 | 3.628E+7 | 3.359E+7 | 2.477E+7 | 7.472E+6 | 3.631E+7 | 3.259E+7 | 1.974E+7 |
|  | $3$ | 1.804E+5 | 1.934E+5 | 1.854E+5 | 1.825E+5 | 1.804E+5 | 3.648E+7 | 3.439E+7 | 2.511E+7 | 1.813E+6 | 3.705E+7 | 3.473E+7 | 2.334E+7 |
|  | 4 | 1.212E+5 | 1.316E+5 | 1.263E+5 | 1.239E+5 | 1.212E+5 | 3.706E+7 | 3.519E+7 | 2.623E+7 | 7.285E+5 | 3.730E+7 | 3.558E+7 | 2.546E+7 |
|  | 5 | 9.286E+4 | 1.018E+5 | 9.784E+4 | 9.580E+4 | 9.286E+4 | 3.749E+7 | 3.609E+7 | 2.778E+7 | 4.047E+5 | 3.752E+7 | 3.641E+7 | 2.698E+7 |

| (B) |  | SD LV 2 → LV 1 | | | | SD LV 2 → LV 0 | | | | SD LV 1 → LV 0 | | | |
| --- | --- | --- | --- | --- | --- | --- | --- | --- | --- | --- | --- | --- | --- |
| $\tau$ | $C_{\nu}$ | N/A | 60% | 70% | 80% | N/A | 60% | 70% | 80% | N/A | 60% | 70% | 80% |
| 0.79 | $1$ | 8.843E+5 | 8.844E+5 | 8.842E+5 | 8.843E+5 | 8.843E+5 | 2.358E+7 | 1.902E+7 | 8.521E+6 | 1.690E+7 | 1.695E+7 | 1.690E+7 | 1.690E+7 |
|  | $2$ | 2.191E+5 | 2.190E+5 | 2.190E+5 | 2.191E+5 | 2.191E+5 | 2.085E+7 | 2.003E+7 | 1.210E+7 | 2.635E+6 | 2.402E+7 | 1.860E+7 | 1.164E+7 |
|  | $3$ | 1.205E+5 | 1.201E+5 | 1.201E+5 | 1.203E+5 | 1.205E+5 | 2.031E+7 | 2.005E+7 | 1.361E+7 | 6.363E+5 | 2.155E+7 | 1.990E+7 | 1.272E+7 |
|  | 4 | 8.416E+4 | 8.364E+4 | 8.372E+4 | 8.392E+4 | 8.416E+4 | 2.022E+7 | 2.008E+7 | 1.462E+7 | 2.831E+5 | 2.063E+7 | 2.006E+7 | 1.386E+7 |
|  | 5 | 6.565E+4 | 6.504E+4 | 6.512E+4 | 6.531E+4 | 6.565E+4 | 2.019E+7 | 2.010E+7 | 1.536E+7 | 1.687E+5 | 2.036E+7 | 2.010E+7 | 1.473E+7 |
| 0.6 | $1$ | 1.819E+6 | 1.853E+6 | 1.821E+6 | 1.819E+6 | 1.819E+6 | 3.607E+7 | 3.250E+7 | 2.303E+7 | 2.276E+7 | 2.311E+7 | 2.276E+7 | 2.276E+7 |
|  | $2$ | 3.583E+5 | 3.784E+5 | 3.635E+5 | 3.598E+5 | 3.583E+5 | 3.628E+7 | 3.359E+7 | 2.478E+7 | 7.473E+6 | 3.631E+7 | 3.259E+7 | 2.470E+7 |
|  | $3$ | 1.804E+5 | 1.936E+5 | 1.856E+5 | 1.826E+5 | 1.804E+5 | 3.648E+7 | 3.439E+7 | 2.519E+7 | 1.814E+6 | 3.705E+7 | 3.473E+7 | 2.513E+7 |
|  | 4 | 1.212E+5 | 1.317E+5 | 1.264E+5 | 1.239E+5 | 1.212E+5 | 3.706E+7 | 3.519E+7 | 2.629E+7 | 7.293E+5 | 3.730E+7 | 3.558E+7 | 2.578E+7 |
|  | 5 | 9.286E+4 | 1.019E+5 | 9.789E+4 | 9.584E+4 | 9.286E+4 | 3.749E+7 | 3.609E+7 | 2.779E+7 | 4.051E+5 | 3.752E+7 | 3.641E+7 | 2.703E+7 |

# The vaccination effects on SARS-CoV-2 variants

**
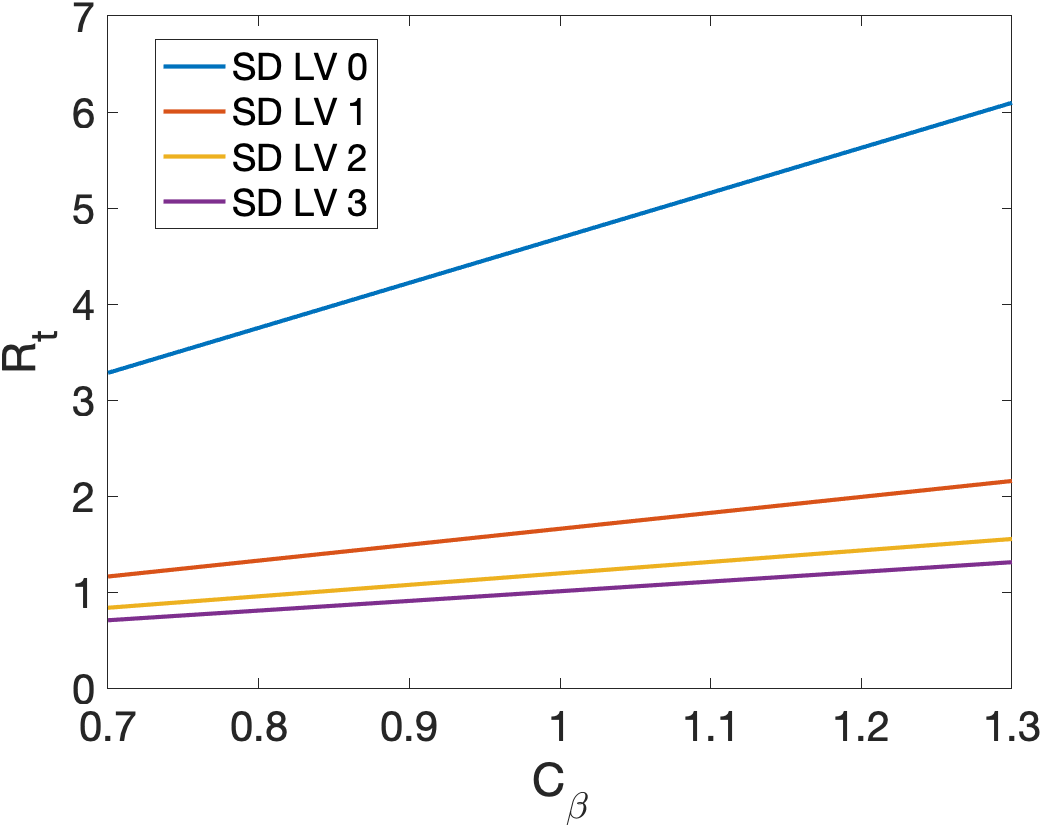
**

Figure 6. Plot of $\mathbf{R}_{\mathbf{t}}$ corresponding to $\boldsymbol{\beta}$ that is varied as $\boldsymbol{\beta\times}\mathbf{C}_{\boldsymbol{\beta}}$for $\mathbf{C}_{\boldsymbol{\beta}}\mathbf{=0.7 - 1.3}$ under SD LV 0, 1, 2, and 3, and the rollout speed $\mathbf{C}_{\boldsymbol{\nu}}\mathbf{=1, 2, 3, 4}$.


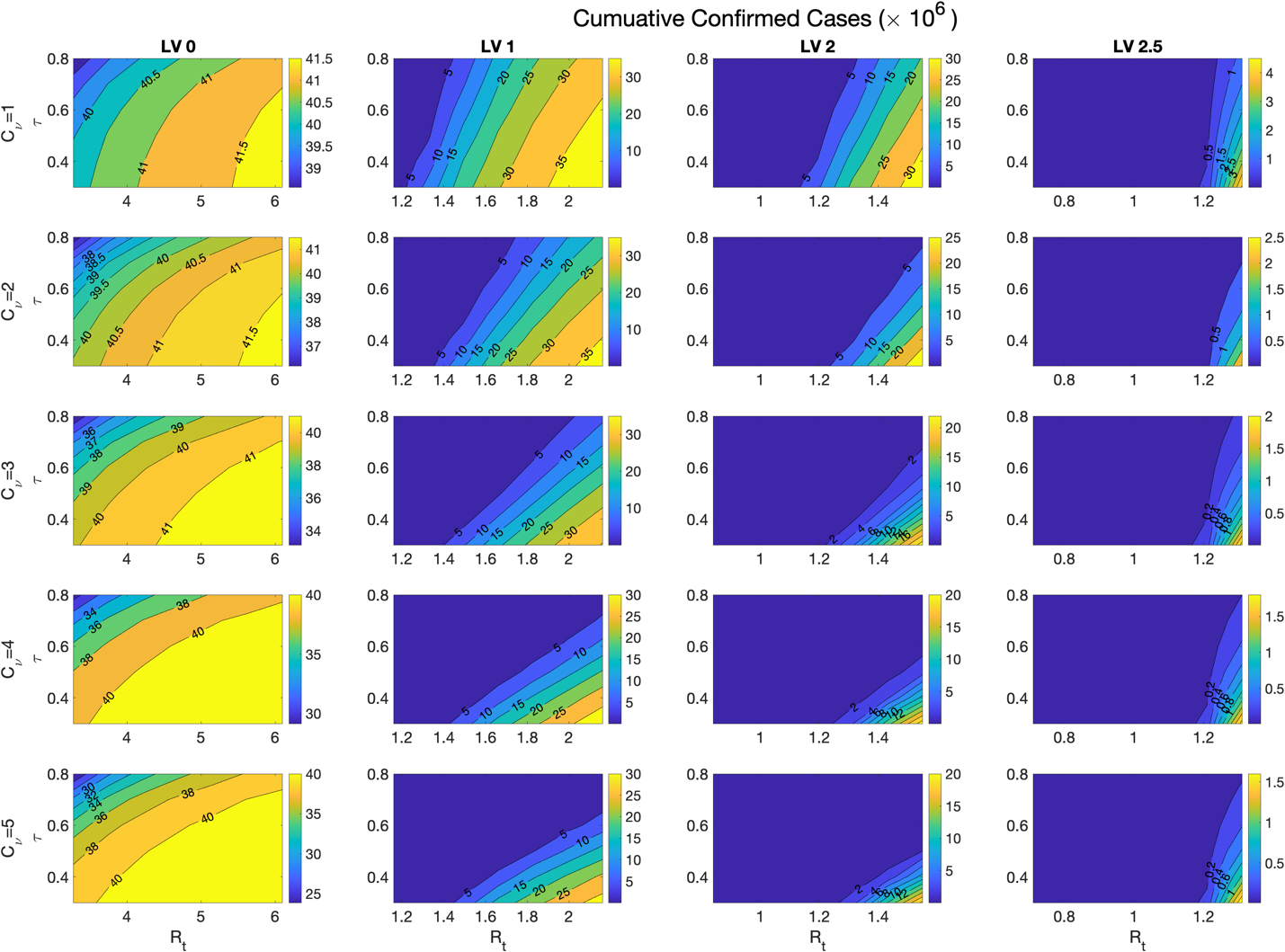


Figure 7. Cumulative confirmed cases $\boldsymbol{(\times}\boldsymbol{10}^{\boldsymbol{6}}\boldsymbol{)}$ for $\boldsymbol{\tau=0.3 - 0.8}$ and for $\boldsymbol{R}_{\boldsymbol{t}}$ corresponding to $\boldsymbol{\beta}$ that is varied as $\boldsymbol{\beta\times}\boldsymbol{C}_{\boldsymbol{\beta}}$for $\boldsymbol{C}_{\boldsymbol{\beta}}\boldsymbol{=0.7 - 1.3}$ under SD LV 0, 1, 2, and 3, and the rollout speed $\boldsymbol{C}_{\boldsymbol{\nu}}\boldsymbol{=1, 2, ...,5}$.


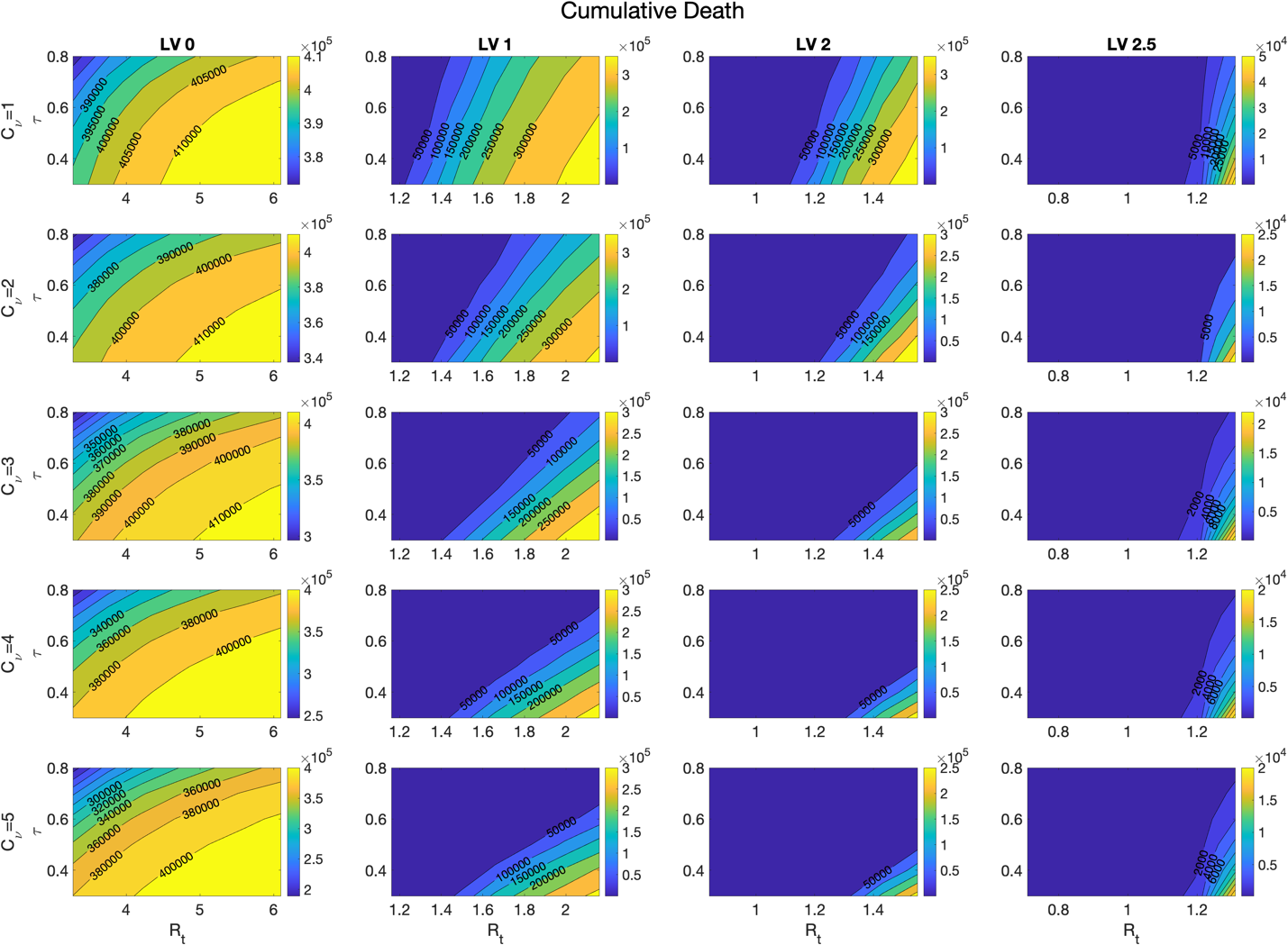


Figure 8. Cumulative deaths for $\boldsymbol{\tau=0.3 - 0.8}$ and for $\boldsymbol{R}_{\boldsymbol{t}}$ corresponding to $\boldsymbol{\beta}$ that is varied as $\boldsymbol{\beta\times}\boldsymbol{C}_{\boldsymbol{\beta}}$for $\boldsymbol{C}_{\boldsymbol{\beta}}\boldsymbol{=0.7 - 1.3}$ under SD LV 0, 1, 2, and 3, and the rollout speed $\boldsymbol{C}_{\boldsymbol{\nu}}\boldsymbol{=1, 2, ...,5}$.


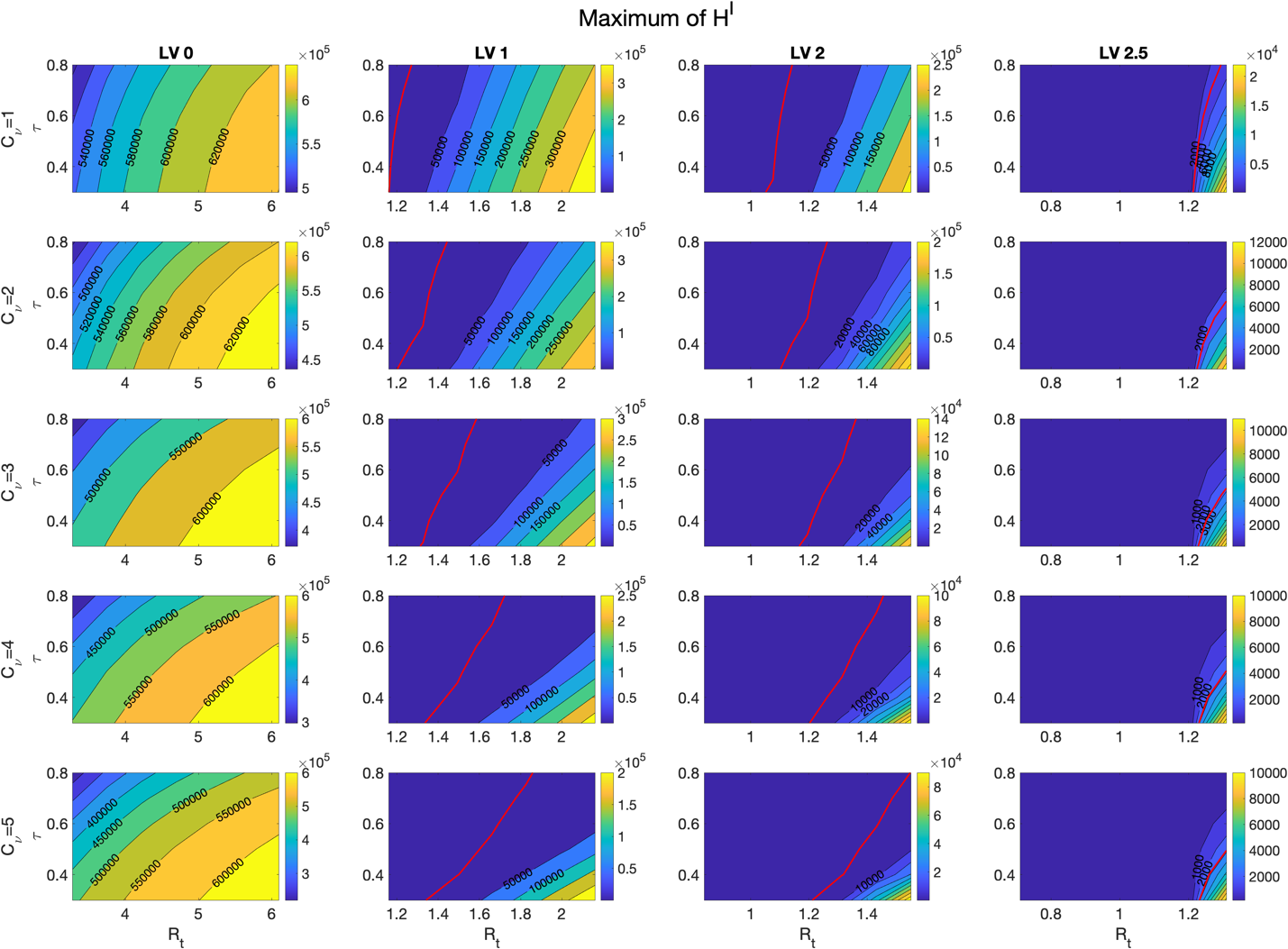


Figure 9. Maximum of hospitalized population with severe symptoms for $\boldsymbol{\tau=0.3 - 0.8}$ and for $\boldsymbol{R}_{\boldsymbol{t}}$ corresponding to $\boldsymbol{\beta}$ that is varied as $\boldsymbol{\beta\times}\boldsymbol{C}_{\boldsymbol{\beta}}$for $\boldsymbol{C}_{\boldsymbol{\beta}}\boldsymbol{=0.7 - 1.3}$ under SD LV 0, 1, 2, and 3, and the rollout speed $\boldsymbol{C}_{\boldsymbol{\nu}}\boldsymbol{=1, 2, ...,5}$. Red lines indicate 2800, which is the capacity of the intensive care unit for COVID-19 patients in Korea.
